# Effects of high-intensity intermittent exercise on glucose and lipid metabolism in type 2 diabetes patients: a systematic review and meta-analysis

**DOI:** 10.3389/fendo.2024.1360998

**Published:** 2024-06-24

**Authors:** Jingwen Feng, Qiuhua Zhang, Baoyi Chen, Jinping Chen, Wenjun Wang, Yuhang Hu, Jiabin Yu, Huiming Huang

**Affiliations:** ^1^ Faculty of Sports Science, Research Academy of Grand Health, Ningbo University, Ningbo, Zhejiang, China; ^2^ NanJing MaiGaoQiao Community Health Service Center, Nanjing, Jiangsu, China; ^3^ Nanjing Kuanyue Health Technology Co., Ltd, Nanjing, Jiangsu, China; ^4^ Ningbo New Fitness Health Technology Co., Ltd, Zhejiang, Ningbo, China

**Keywords:** high-intensity interval exercise, type 2 diabetes mellitus, glucose metabolism, lipid metabolism, meta-analysis

## Abstract

**Objective:**

To evaluate the effects of high-intensity interval training (HIIT) on glycolipid metabolism among type 2 diabetes patients.

**Methods:**

HIIT is consistent with an exercise program (65%-90%VO_2_max or 75%-95% HRmax; exercise cycle≥2 weeks; frequency ≥ 2 times/week). A meta-analysis was conducted utilizing the random effects model to synthesize the data.

**Results:**

A total of 22 RCT studies with 1034 diabetic patients were included. Compared to moderate-intensity aerobic exercise or conventional controls, HIIT yields noteworthy effects on FBG (MD: -0.55; 95% CI: -0.85- -0.25, Hedges’ g =0.98), 2h-PG (MD: -0.36; 95% CI: -0.57- -0.14, Hedges’ g =1.05), FINS (MD: -0.41; 95% CI: -0.79- -0.03, Hedges’ g =1.07), HbA1c (MD: -0.60; 95% CI: -0.84- -0.36, Hedges’ g =2.69), TC (MD: -0.58; 95% CI: -0.80- -0.36, Hedges’ g =2.36), TG (MD: -0.50; 95% CI: -0.86- -0.14, Hedges’ g =1.50), HDL (MD: 0.62; 95% CI: 0.29–0.95, Hedges’ g =1.19) and LDL (MD: -0.31; 95% CI: -0.56- -0.08, Hedges’ g =0.91), all of the above p<0.01.

**Conclusions:**

HIIT has been shown to improve glucose and lipid metabolism in patients with type 2 diabetes, especially in HbA1c, TC, TG, and HDL. For patients between the ages of 40 and 60 with less than 5 years of disease, exercise programs of moderate to longer duration or moderate to high intensity will produce more favorable results.

## 1 Introduction

Type 2 diabetes mellitus (T2DM), a highly prevalent chronic metabolic disorder, is the most commonly observed variant of diabetes. It is distinguished by elevated blood glucose levels, relative insufficiency of insulin, and resistance to insulin (1). Abnormal blood glucose levels are commonly accompanied by dyslipidemia or hypertension, resulting in both incapacitation and reduced life expectancy for affected individuals, as well as an elevated susceptibility to sudden cardiac death (2, 3). Studies have shown that diabetes contributes to an annual global mortality of around 3 million individuals, with a consistent increase in the global prevalence of diabetes each year (1). Consensus reports from the American Diabetes Association (ADA) and the European Association for the Study of Diabetes (EASD) provide updated strategies for the management of type 2 diabetes in adults, proposing person-centered, holistic care that carefully considers the preferences of the person with diabetes to inform the individualization of treatment goals and strategies (3).

Regular aerobic exercise is a routine treatment option for addressing diabetes mellitus, and aerobic training can reduce glycated hemoglobin levels, increase maximal oxygen uptake, and improve insulin sensitivity in patients with T2DM (4). Nevertheless, aerobic exercise takes a long time. American Sports Medicine Association (ACSM) exercise guidelines recommend aerobic exercise for people with diabetes up to 300 min per week (5). Some studies have pointed out that lack of time and low interest in exercise are the primary barriers to physical activity (4, 6). In practical terms, most patients do not fully engage in this self-intervention approach. High-intensity interval training (HIIT) represents a novel exercise regimen comprising multiple high- intensity training and low- intensity training intervals, which significantly reduces the exercise duration to achieve the same effect as aerobic exercise and avoids the appearance of uncomfortable symptoms during the low intensity intervals (6). Reindell suggested that HIIT has the characteristics of a short time, high efficiency, and favorable outcomes. In comparison to other exercise interventions, it can utilize a shorter time to achieve the same training effect (7). Khurshid et al. found that both short-term high-intensity exercise patterns and evenly distributed exercise patterns reduced the risk of cardiovascular disease, with the former being more feasible (8).

The current findings on the effects of HIIT on glucose and lipid metabolism in type 2 diabetic populations are inconsistent. Liu et al. showed that HIIT was superior to moderate-intensity continuous training (MICT) in reducing blood glucose and lipid indexes; however, no significant difference was observed in lowering 2-hour postprandial glucose levels or improving HbA1c levels (9). Han’s study similarly noted that HIIT is ineffective in terms of improvement in FPG, HbA1c, and lipid metabolism (10). In addition, some studies still have limitations and lack analysis in terms of the biological characteristics of the patients and the subgroup characteristics of the exercise protocol. Liu’s review explored the effects of HIIT on glycemia, cardiorespiratory fitness, and body composition, but no subgroup analyses were performed (9). Chen et al.’s review was published in Chinese, and the study population was mainly Chinese (6). Ivan et al. included studies in English and Spanish before 2017, with a small number of documents, after which some new evidence appeared (11); Yang reviewed the effects of low-intensity HIIT on glucose metabolism and cardiorespiratory endurance in diabetic patients, who showed significant improvements in glycemic control, insulin resistance, and lipids. However, subgroup analyses were not performed due to a smaller number of included studies (12). Jung ME et al. reported that despite patients’ knowledge that exercise is effective, a lack of scientific guidance resulted in decreased adherence to exercise among diabetic patients (13). To enhance patient engagement in self-care practices that can be tailored to individual preferences and traits, further investigation into the various factors that influence exercise regimens is necessary for optimization.

Therefore, we conducted a meta-analysis and systematic review to verify the effects of HIIT on glycemic and lipid markers considering its duration, intensity and aspects related to the disease and sociodemographic factors in the analysis. The results enrich its proposal and can contribute to decision making for prescribing training to patients with type 2 diabetes.

## 2 Materials and methods

### 2.1 Registration

The protocol has been registered in the PROSPERO registry (CRD42023401649; http://www.crd.york.ac.uk/PROSPERO) and conducted in accordance with the systematic review checklist (PRISMA 2020).

### 2.2 Literature search strategy

Literature searches were performed in the PubMed, Scopus, Web of Science, Cochrane Library, and China National Knowledge Infrastructure(CNKI) databases (details of the search strategies are reported in **Table 1**).

**Table 1:** Systematic literature review search terms and strategy.

Search terms for PubMed
#1 “2 type diabetes”[MeSH Terms] OR “2 type diabetes”[All Fields] OR “diabetes”[MeSH Terms] OR “diabetes”[All Fields]
#2 “High Intensity Interval Training”[MeSH Terms] OR “High Intensity Interval Training”[All Fields] OR “HIIT”[MeSH Terms] OR “HIIT”[All Fields] OR “Sprint Interval Training”[MeSH Terms] OR “Sprint Interval Training”[All Fields]
#3 “blood glucose”[MeSH Terms] OR “blood glucose”[All Fields] OR “Blood lipids”[MeSH Terms] OR “Blood lipids”[All Fields] OR “Impaired glucose tolerance”[MeSH Terms] OR “Impaired glucose tolerance”[All Fields]
#4 systematic OR Meta-Analysis
#5 #1 AND #2
#6 #3 AND #5
#7 #6 NOT #4
Search terms for Cochrane library
#1 (2 type diabetes):ti,ab,kw OR (diabetes):ti,ab,kw (Word variations have been searched)
#2 (High Intensity Interval Training):ti,ab,kw OR (Sprint Interval Training):ti,ab,kw OR (HIIT):ti,ab,kw (Word variations have been searched)
#3 (blood glucose):ti,ab,kw OR (blood lipids):ti,ab,kw OR (Impaired glucose tolerance):ti,ab,kw (Word variations have been searched)
#4 #1 and #2
#5 #3 and #4 (restricted as Cochrane Reviews or other reviews)
Search terms for Web of science
TS=((Diabetes Mellitus, Noninsulin-Dependent OR 2 type diabetes OR diabetes) AND (Sprint Interval Training OR High Intensity Interval Training OR HIIT) AND (blood glucose OR Impaired glucose tolerance OR Blood lipids))
Search terms for Scopus
#1TITLE-ABS-KEY(“Diabetes Mellitus, Noninsulin-Dependent’’ OR “2 type diabetes’’ OR “diabetes”)
#2TITLE-ABS-KEY(“Sprint Interval Training’’ OR “High Intensity Interval Training’’ OR “HIIT”)
#3TITLE-ABS-KEY(“blood glucose’’ OR “Impaired glucose tolerance’’ OR “Blood lipids”)
#1 AND #2 AND #3
Search terms for CNKI
((Diabetes Mellitus, Noninsulin-Dependent OR 2 type diabetes OR diabetes) AND (Sprint Interval Training OR High Intensity Interval Training OR HIIT) AND (blood glucose OR Impaired glucose tolerance OR Blood lipids))

The search dates were all from the creation of the database to April 1, 2023 The scientific databases were searched according to three criteria: study population (“diabetes mellitus”, “type 2 diabetes mellitus”), medical interventions (“high-intensity interval exercise”, “sprint interval training”, “HIIT”) and outcomes (“blood glucose”, “glucose tolerance disorder”, “lipids”). All search strategies were conducted in the relevant databases using English and Chinese. Two researchers independently completed the initial screening of the articles and the statistics of the basic information in the included literature and the changes in the effect indicators before and after the intervention, and the third researcher negotiated the resolution of disputes when they existed.

### 2.3 Inclusion and exclusion criteria

**Figure 1:**
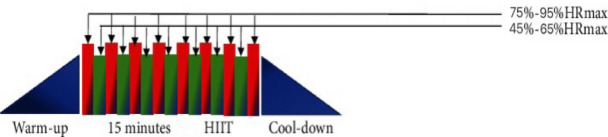
Exercise protocol of HIIT.

The eligibility criteria were established depending on the PICOS (population, intervention, comparison, outcome, study design) items.

(P) The study included patients with type 2 diabetes who met the World Health Organization’s diagnostic criteria (fasting blood glucose≥7.0 mmol/L or OGTT 2-hour glucose≥11.1 mmol/L or HbA1C≥6.5%), and who had an age≥18 years; there were no restrictions on the gender or race of the study participants. Patients with clinically manifest cardiovascular diseases, acute complications, and pregnant or lactating women were excluded.

(I) The intervention group participated in only the HIIT exercise program. HIIT exercise protocol consists of three phases: a warm-up phase, an alternating exercise phase, and an exercise recovery phase (13). The alternating phase alternates high-intensity exercise with low-intensity exercise (the exercise protocol of HIIT is shown in **Figure 1**). During the high-intensity exercise phase, the heart rate should be 75%-95% of the HRmax for 60 s, and during the low-intensity interval phase, the heart rate should be 45%-65% of the HRmax for 60 s. A total of 6–8 sets were completed; HIIT is a form of exercise that can be practiced in a variety of ways (exercise cycle ≥ 2 weeks; frequency ≥ 2 times/week). Exercise types include running, cycling, resistance bands, and unassisted exercise. Studies on joint interventions by combining strength training, diet, medicine, health education, and other means were excluded.

(C) The comparators included moderate-intensity continuous training (MICT, 46%-63% VO2max or 64%-75% HRmax; a duration of usually more than 20 min; types such as running, cycling, walking, body mechanics, and Tai Ji), combined aerobic and resistance training, routine care groups, and static stretching. Studies with control groups practicing low- or moderate-intensity HIIT were excluded.

(O) The outcomes included any of the following indicators. Primary outcomes: fasting blood glucose, glycosylated hemoglobin, fasting insulin, and 2h-PG; secondary outcomes: total cholesterol, triglyceride, high-density lipoprotein, and low-density lipoprotein. Studies that did not contain relevant outcome indicators were excluded.

(S) The study design included randomized controlled trials. Case reports, abstracts, reviews, lectures, commentaries, and data that could not be extracted were excluded.

### 2.4 Evaluation of bias and quality assessment

We used the Cochrane Quality Assessment Tool, as the included articles were randomized controlled trials. The risk of bias and methodological quality of the included studies were assessed by two evaluators using Review Manager 5.3 in terms of selective bias (randomized sampling, grouping), implementation bias (whether the experiment was blinded to the subjects and experimenters), measurement bias (whether the experiment operator was blinded to the endpoints), follow-up bias (completeness of the results), selective reporting bias and other biases were evaluated. The outcomes were expressed as low- risk, unclear, and high- risk. If a dispute arose between the two evaluators during the quality evaluation, a third evaluator was invited to participate to reach harmonization.

### 2.5 Data extraction

After screening the literature, two researchers independently extracted the following data from the eligible literature: the external characteristics of the literature (title, authors, year of publication, nationality of the authors); basic information about the subjects (age, gender, country, sample size, duration of the disease); the experimental design and exercise intervention protocol (training period and frequency, duration, intervention mode, intensity); and the outcome indicators related to the study.

The subgroups were the intensity of exercise (75%-79% HRmax, 80%-89% HRmax and >=90%, HRmax), exercise period (≤8 weeks, 9–12 weeks, and >12 weeks), exercise duration (≤30 min/time and >30 min/time), age (<40 years, 40–59 years, and ≥60 years), and disease duration (<5 years, 5–10 years, and >10 years). In cases of disagreement between the two persons, a third person summarized them and determined their subgroups through a group discussion. The outcome indicators need to be extracted separately for the mean and standard deviation of the pre-test and the mean and standard deviation of the post-test for the intervention and control groups in the study. The mean standard deviation of the difference between the pre-test and post-test was calculated separately.

For some studies (14–24), as they had multiple control groups, we extracted the data across multiple groups and included them in the meta-analysis (**Table 2**). For FPG, we evaluated 14 studies, of which 21 compared HIIT and control groups. For 2h-PG, 9 studies were evaluated, with 15 comparisons between the HIIT and control groups. For FINS, 8 studies were evaluated, with 14 comparisons between the HIIT and comparison groups. For HbA1c, 18 studies were evaluated, with 30 comparisons between the HIIT and control groups. For TC, 13 studies were evaluated, with 23 comparisons between the HIIT and control groups. For TG, 13 studies were evaluated, and 23 comparisons were made between the HIIT and comparison groups. For HDL, 15 studies were evaluated, with 24 comparisons between the HIIT and control groups. For LDL, 15 studies were evaluated, with 24 comparisons between the HIIT and comparison groups.

**Table 2:** characteristics of the included literature.

Study	Country, Study design	Sample size	Age (years)	Medications		Disease duration (years)		Intervention characteristics					Comparator	Outcomes	Drop out
				Metformin	Statins			Type	Duration (Weeks)	Intensity	Frequency (times/week)	Average Duration (min/time)			
Wu,2022 (15)(1)	China RCT	EG: 23 CG: 21	EG: 54 CG: 57	EG: 23 CG: 21	EG: 23 CG: 21	EG: CG:	7 6	cycling	12	Moderate	3	20	MICT	①②③ ④⑤⑥⑦⑧	EG:0 CG:0
Wu,2022 (15)(2)	China RCT	EG: 23 CG: 18	EG: 54 CG:56	EG: 23 CG: 18	EG: 23 CG: 18	EG: CG:	7 6	cycling	12	Moderate	3	20	usual care	①②③ ④⑤⑥⑦⑧	EG:0 CG:0
Ren,2022 (25)	China RCT	EG: 16 CG:14	EG: 60.38 CG: 59.71	–	–	EG: CG:	12.93 8.79	unassisted exercise	12	Moderate	2	20	MICT	①②③ ⑤⑥⑦⑧	EG:0 CG:2
Wu,2020 (16)(1)	China RCT	EG: 32 CG: 32	EG: 35.06 CG: 34.56	EG: 32 CG: 32	–	EG: CG:	1.3 1.58	unassisted exercise	12	Moderate	5	21	MICT	①②③ ④⑤⑥⑦⑧	EG:0 CG:0
Wu,2020 (16)(2)	China RCT	EG: 32 CG: 32	EG: 35.06 CG: 34.56	EG: 32 CG: 32	–	EG: CG:	1.3 1.58	unassisted exercise	24	Moderate	5	21	MICT	①②③ ④⑤⑥⑦⑧	EG:0 CG:0
Wang,2019 (26)	China RCT	EG: 34 CG: 31	EG: 48.32 CG: 46.71	–	–	EG: CG:	5.76 5.65	run	12	Light	3	–	MICT	①②③⑦⑧	EG:1 CG:4
Deng,2020 (27)	China RCT	EG: 40 CG: 37	EG: 50.20 CG: 50.80	–	–	EG: CG:	9.60 9.60	run	8	Moderate	3	30	MICT	①②	EG:2 CG:5
Ahmad,2019(1) (17)	Egypt RCT	EG: 8 CG: 9	EG: 35.75 CG: 38	EG: 8 CG: 9	EG: 8 CG: 9	EG: CG:	N\A	run	8	Moderate	3	–	MICT	②	EG: 2 CG: 1
Ahmad,2019(2) (17)	Egypt RCT	EG: 8 CG: 9	EG: 35.75 CG: 41.5	EG: 8 CG: 9	EG: 8 CG: 9	EG: CG:	N\A	run	8	Moderate	3	–	usual care	②	EG: 2 CG: 1
Chénard,2021 (28)	Canada RCT	EG: 14 CG: 15	EG: 63.0 CG: 64.1	EG: 10 CG: 10	–	EG: CG:	5.1 9	run	12	Moderate	3	25	MICT	①②④	EG: 1 CG: 0
Hamidreza,2019 (29)	Iran RCT	EG: 10 CG: 10	EG: 37.80 CG: 37.5	–	–	EG: CG:	N\A	cycling	8	–	3	16	usual care	⑤⑥⑦⑧	EG: 1 CG: 1
Li Jun,2022(1) (18)	China RCT	EG: 13 CG: 12	EG: 38 CG: 39	EG: 13 CG: 12	EG: 13 CG: 12	EG: CG:	1.95 1.79	cycling	12	Moderate	5	15	MICT	①②④	EG: 0 CG: 1
Li Jun,2022(2) (18)	China RCT	EG: 13 CG: 12	EG: 38 CG: 40	EG: 13 CG: 12	EG: 13 CG: 12	EG: CG:	1.95 1.79	cycling	12	Moderate	5	15	usual care	①②④	EG: 0 CG: 1
zadeh,2022(1) (19)	Iran RCT	EG: 17 CG: 18	EG: 52.2 CG: 51.6	–	–	EG: CG:	3 3	cycling	12	High	4	20–30	RT	②③⑤⑥⑦	EG:7 CG:6
zadeh,2022(2) (19)	Iran RCT	EG: 17 CG: 17	EG: 52.2 CG: 52.8	–	–	EG: CG:	3 3	cycling	12	Light	4	20–30	AT	②③⑤⑥⑦	EG:7 CG:7
zadeh,2022(3) (19)	Iran RCT	EG: 17 CG: 16	EG: 52.2 CG: 53.2	–	–	EG: CG:	3 3	cycling	12	–	4	20–30	Combined A+R trainings	②③⑤⑥⑦	EG:7 CG:8
Adalberto, 2020(1) (20)	Brazil RCT	EG: 16 CG: 16	EG: 69.55 CG: 69.55	–	–	EG: CG:	N\A	unassisted exercise	8	–	3	40	MICT	②⑤⑥⑦⑧	EG:0 CG:0
Adalberto, 2020(2) (20)	Brazil RCT	EG: 16 CG: 16	EG: 69.55 CG: 69.55	–	–	EG: CG:	N\A	unassisted exercise	8	–	3	40	Sedentary Control	②⑤⑥⑦⑧	EG:0 CG:0
Ebrahim,2019(1) (21)	Iran RCT	EG: 14 CG: 14	EG: 55.36 CG: 54.14	EG: 14 CG: 13	–	EG: CG:	N\A	cycling	10	High	3	50	Combined A+R trainings	①②④	EG:3 CG:3
Ebrahim,2019(2) (21)	Iran RCT	EG: 14 CG: 14	EG: 55.36 CG: 55.71	EG: 14 CG: 13	–	EG: CG:	N\A	cycling	10	High	3	50	usual care	①②④	EG:3 CG:4
Sophie,2016 (30)	N\A	EG: 12 CG: 11	EG: 61 CG: 59	EG: 7 CG: 7	EG: 7 CG:6	EG: CG:	5 4	resistance band	12	–	3	N\A	usual care	①②③ ④	EG:2 CG:1
Sophie,2019 (31)	UK RCT	EG: 11 CG:11	EG: 59 CG: 60	EG: 7 CG: 7	EG: 7 CG:6	EG: CG:	5 4	resistance band	12	–	3	N\A	usual care	②	EG:3 CG:3
Mostafa, 2021(1) (14)	Iran RCT	EG: 16 CG: 13	EG: 52.02 CG: 52.28	EG: 10 CG: 8	EG: 9 CG: 7	EG: CG:	7.25 6.76	cycling	16	Moderate	2	N\A	usual care	①②④⑤⑥⑦⑧	EG:0 CG:0
Mostafa, 2021(2) (14)	Iran RCT	EG: 16 CG: 13	EG: 52.02 CG: 51.31	EG: 10 CG: 11	EG: 9 CG:6	EG: CG:	7.25 6.76	cycling	16	Moderate	2	N\A	RT	①②④⑤⑥⑦⑧	EG:0 CG:0
F. Maillard, 2016 (32)	France RCT	EG: 8 CG: 9	EG: 68.2 CG: 70.1	EG: 8 CG: 9	EG: 8 CG: 9	EG: CG:	14.5 14.5	cycling	16	Light	2	20	MICT	①②⑤⑥⑦⑧	EG:0 CG:1
Gulin, 2023(1) (24)	N\A	EG: 21 CG: 21	EG: 57.5 CG: 55.42	EG: 21 CG: 21	–	EG: CG:	1–10	cycling	12	Moderate	3	48	MICT	②⑤⑥⑦⑧	EG:1 CG:1
Gulin, 2023(2) (24)	N\A	EG: 21 CG: 21	EG: 57.5 CG: 55.75	EG: 21 CG: 21	–	EG: CG:	1–10	cycling	12	Moderate	3	48	Static Stretching	②⑤⑥⑦⑧	EG:1 CG:1
liu,2019 (9)	China RCT	EG: 93 CG: 90	EG: 67.89 CG: 68.82	–	–	EG: CG:	9.79 10.38	run	8	Moderate	3	20	MICT	①③⑤⑥⑦⑧	EG: 4 CG: 9
Chénard,2021 (28)	Canada	EG: 14 CG: 15	EG: 67.0 CG: 68.3	EG: 10 CG: 10	EG: 12 CG: 10	EG: CG:	10.4 9.2	run	12	Moderate	3	25	MICT	⑤⑥⑦⑧	EG: 1 CG: 0
C. Alvarez, 2016 (33)	Chile RCT	EG: 13 CG: 10	EG: 45.6 CG: 43.1	–	–	EG: CG:	3.4 3.6	run	16	High	3	30	usual care	②⑤⑥⑦⑧	EG: 1 CG: 4
Kamilla M., 2017(1) (23)	Denmark RCT	EG: 13 CG: 12	EG: 54 CG: 58	EG: 12 CG: 10	–	EG: CG:	8 6	cycling	11	High	3	20	MICT	①②③ ④⑤⑥⑦⑧	EG: 1 CG: 0
Kamilla M., 2017(2) (23)	Denmark RCT	EG: 13 CG: 7	EG: 54 CG: 57	EG: 12 CG: 6	–	EG: CG:	8 7	cycling	11	High	3	20	usual care	①②③ ④⑤⑥⑦⑧	EG: 1 CG: 1
Kristian Karstoft, 2013(1) (22)	Denmark RCT	EG: 12 CG: 12	EG: 57.5 CG: 60.8	EG: 7 CG: 7	–	EG: CG:	3.5 6.2	walk	17	Light	5	60	CWT	①②③ ④⑤⑥⑦⑧	EG: 1 CG: 1
Kristian Karstoft, 2013(2) (22)	Denmark RCT	EG: 12 CG: 8	EG: 57.5 CG: 57.1	EG: 7 CG: 3	–	EG: CG:	3.5 4.5	walk	17	Light	5	60	usual care	①②③ ④⑤⑥⑦⑧	EG: 1 CG: 0

RCT, Randomized Controlled trial; EG, experiment group; CG, Control Group; RT, Resistance training; AT, aerobic training; Combined A+R trainings, Combined aerobic+resistance training; CWT, continuous-walking training; moderate intensity: 80%-89%HRmax, light intensity: 75%-79%HRmax, high intensity: >=90%HRmax; MICT, Moderate-Intensity Continuous Training.

Outcomes: ①:FPG ②:HbA1c ③:2h-PG ④:FINS ⑤:TG ⑥:TC ⑦:HDL ⑧:LDL.

### 2.6 Sensitivity analysis and publication bias

To explore the heterogeneity, we conducted a sensitivity analysis, applying a literature-by-exclusion approach. Publication bias was assessed with a visual inspection of a funnel plot. When significant bias was detected, we performed a trim-and-fill analysis.

### 2.7 Statistical analysis

Stata 16 software was used for meta-analysis. The data included in the study were continuous and were expressed as the mean difference (MD) and 95% confidence interval (CI). The presence of heterogeneity among the studies was tested using the I2 test. Subgroup analyses were performed to analyze the characteristics of the studies and sources of heterogeneity across various classifications.

To reflect the practical value of the effect sizes for clinical purposes, the effect size was calculated according to Hedge s’ g. It is also widely used in meta-analysis. Hedges suggested that g values of 0.2, 0.5, and 0.8 represent small, medium, and large effect sizes, respectively (34).

## 3 Results

### 3.1 Description of studies

**Figure 2:**
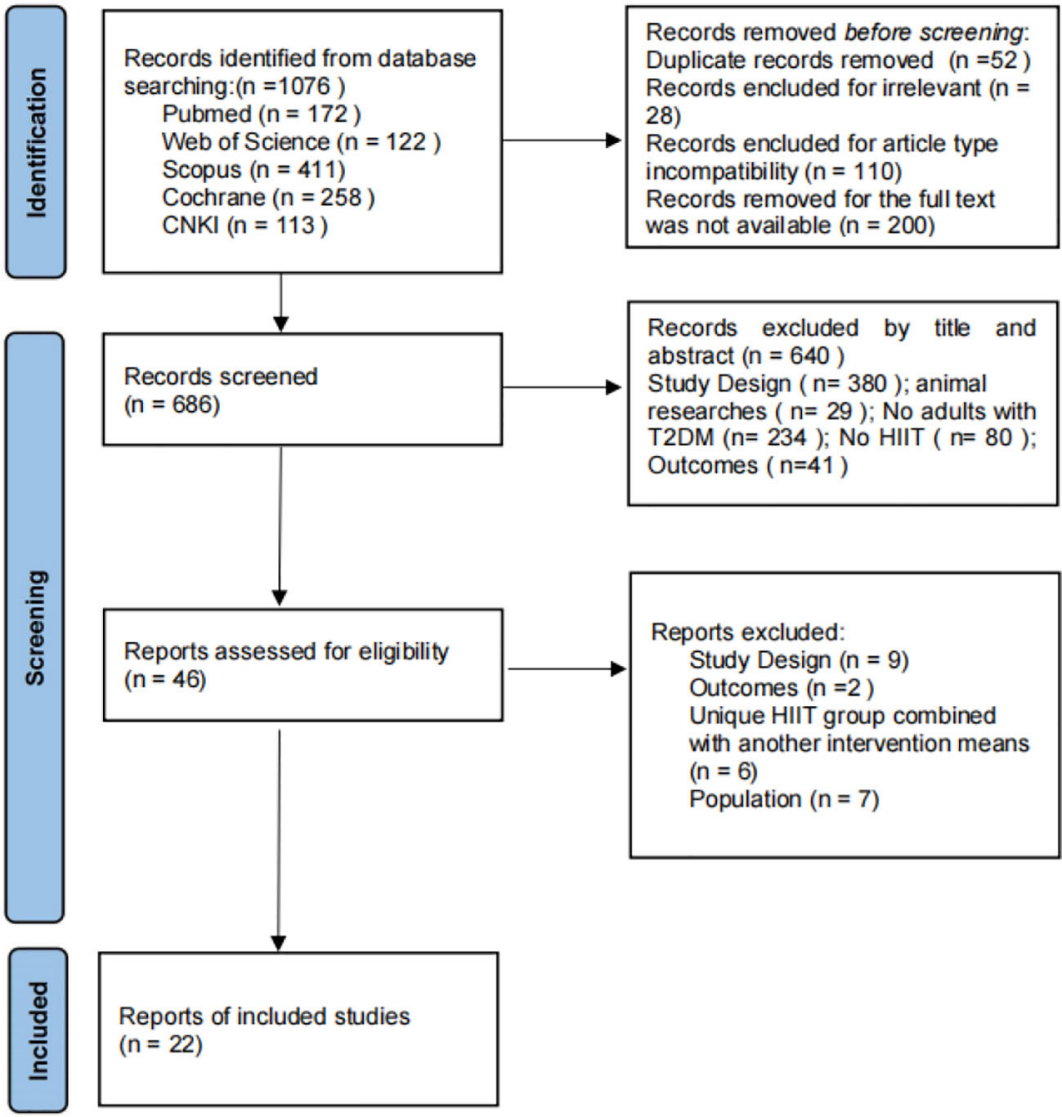
Flowchart of the study selection process.

Through the search strategy, a total of 1076 articles were initially retrieved from 5 databases, and 390 articles that were duplicates or for which the full text could not be accessed were deleted. After reading the article titles and abstracts, 640 articles were excluded, and the full text of the remaining 46 articles was read and screened according to the inclusion and exclusion criteria, resulting in the inclusion of 22 articles. The specific flow of the included studies is shown in **Figure 2**.

This study encompassed RCTs exclusively as part of its research design, with all subjects belonging to the type 2 diabetic population, totaling 1268 patients. The experimental group received interventions involving HIIT exercise, which included running (n=8) (17, 26–28, 33, 35, 36), cycling (n=23) (15, 17, 27, 29–31, 35, 37, 38), unassisted exercise (n=5) (16, 20, 25), resistance bands (n=2) (30, 31), and walking (n=2) (22). Meanwhile, the control group was based on aerobic exercise and usual care. The exercise intensity was categorized as moderate in 17 studies, low in 5 studies, and high in 6 studies. The primary outcome indicators were addressed in 32 studies. Details of the basic characteristics of the included literature are shown in **Table 2**.

### 3.2 Quality evaluation

**Figure 3:**
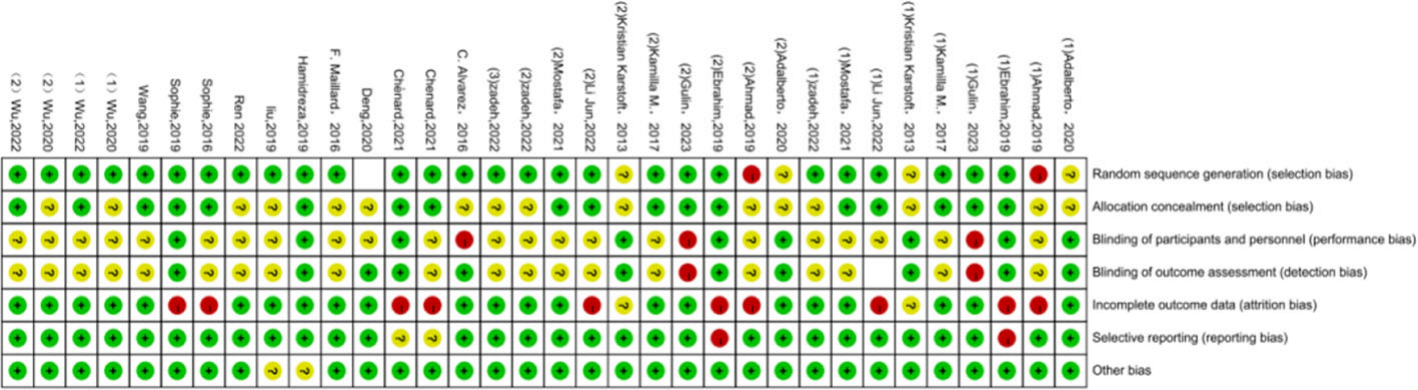
Schematic diagram of methodological quality assessment of literature.

**Figure 4:**
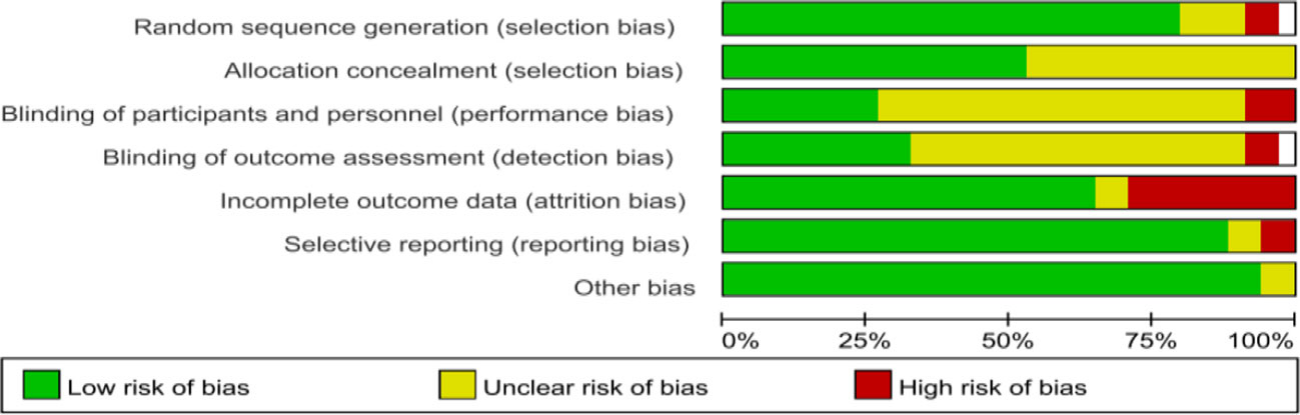
Statistics of each risk factor as a percentage of all included literature.

Regarding the quality assessment, 13 studies were identified as having a high risk, and the results are shown in **Figures 3**, **4**. In **Figure 3**, green means “low risk”, red means “high risk”, and yellow means “unclear”. In **Figure 4**, green means “low risk”, red means “high risk”, and yellow means “unclear”. The results were as follows: (1) randomization— 2 studies had high risk, and 4 did not describe the random allocation method; (2) allocation concealment, 16 studies did not describe the specific allocation method; (3) blinding— 3 studies did not blind the subjects, 2 did not blind the evaluators, and most of the studies did not describe the method of blinding; (4) incomplete data reporting— 9 studies had case dropout, and 2 did not mention the number of subjects at the beginning; (5) reporting bias— 2 papers had case dropout, and 2 did not mention the number of starting subjects; and (6) the results were as follows: (1) the randomization method was not described. In reporting bias, two papers had biases.

### 3.3 Primary outcomes (glucose metabolism)

**Figure 5:**
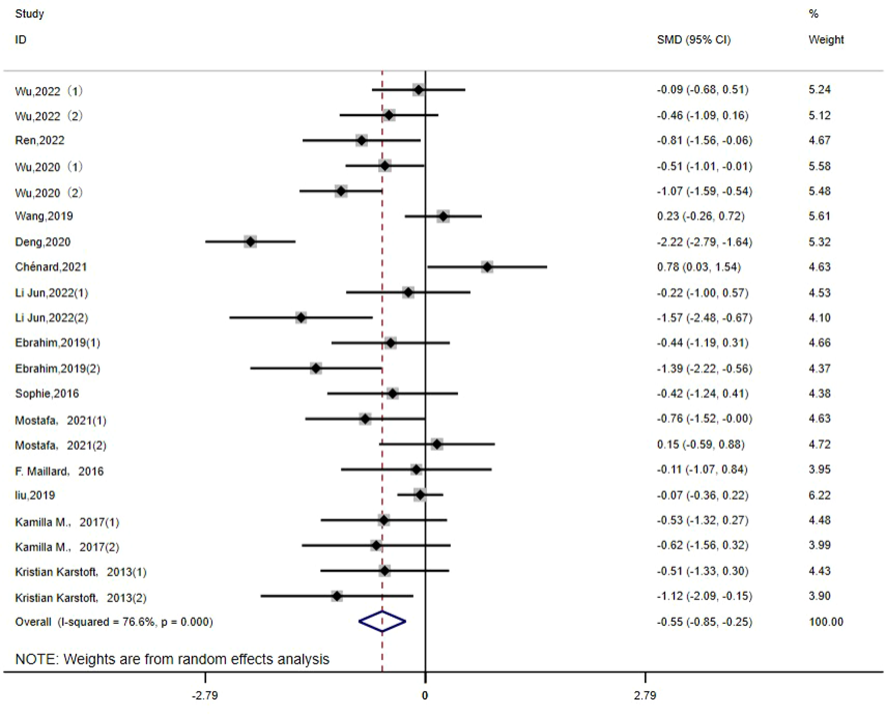
FPG.

FPG: There are 21 studies (14–16, 18, 21–23, 25–28, 30, 32, 36) that report the effect of HIIT on the FGB index in a type 2 diabetic population. The results show that HIIT had a large effect size on FGB (MD: -0.55; 95% CI: -0.85- -0.25; Hedges’ g = 0.98; p < 0.01). The left side of the center line is “in favor of HIIT”(**Figure 5**). Because the I^2^ was 76.6%, a random-effects model was chosen for analysis. Notably, there were notable disparities in the exercise duration and disease duration among the subgroups (**Table 3**), with effect size values for exercise durations of 30 minutes or less surpassing those for durations exceeding 30 minutes (p < 0.01). Furthermore, individuals with a disease duration of less than 5 years (p<0.01) exhibited a significant effect size in terms of reducing FPG. Conversely, no significant differences were observed in the subgroups based on the exercise period or exercise intensity (p=0.267, p=0.194), and the results of fasting glucose were not affected by them.

**Table 3:** Subgroup analysis of glucose metabolism.

Group standard	Study quantity	Mean difference (95% CI)	Hedges’ g	P within group	Heterogeneity		
					P heterogeneity	I2 (%)	P between sub-groups
FBG							
Overall	21	-0.55(-0.85- -0.25)	0.98	<0.01	<0.01	76.6	
Weeks							
≤8	2	-0.51(-0.76- -0.25)	0.18	<0.01	<0.01	97.7	0.267
8–12	13	-0.36(-0.55- -0.17)	1.07	<0.01	<0.01	60.5	
>12	6	-0.64(-0.94- -0.34)	1.33	<0.01	0.098	46.1	
Training intensity,%							
75–79	4	-0.15(-0.50–0.21)	0.34	0.422	0.074	56.7	0.194
80–89	12	-0.48(-0.64- -0.32)	1.39	<0.01	<0.01	84.4	
≥90	4	-0.73(-1.14- -0.32)	1.06	<0.01	0.352	8.3	
Disease duration, year							
<5	5	-0.90(-1.23–0.56)	1.59	<0.01	0.166	38.2	<0.01
5–10	11	-0.29(-0.46–0.12)	0.78	<0.01	<0.01	84.0	
>10	3	-0.53(-0.91–0.14)	0.81	<0.01	0.532	0.0	
Average Duration, min/time							
≤30	13	-0.50(-0.66–0.33)	1.47	<0.01	<0.01	82.4	0.016
>30	4	-0.82(-1.24–0.41)	1.22	<0.01	0.298	18.5	
FINS							
Overall	14	-0.41(-0.79- -0.03)	1.07	0.033	<0.01	68.8	
Training intensity,%							
75–79	2	-2.74(-3.61–1.87)	2.79	<0.01	0.047	74.6	<0.01
80–89	7	-0.08(-0.35–0.18)	0.27	0.548	0.661	0.0	
≥90	4	-0.21(-0.61–0.17)	0.27	0.283	0.941	0.0	
Age, year							
<40	2	-0.16(-0.72- 0.40)	0.48	0.572	0.951	0.0	0.447
40–60	10	-0.35(-0.59–0.10)	1.04	<0.01	<0.01	77.4	
≥60	2	0.01(-0.53–0.55)	0.04	0.972	0.582	0.0	
Average Duration, min/time							
≤30	7	-0.02(-0.29- 0.26)	0.14	0.904	0.935	0.0	<0.01
>30	4	-0.88(-1.33–0.43)	1.06	<0.01	<0.01	89.5	
2hPG							
Overall	15	-0.31(-0.46- -0.16)	1.05	<0.01	0.042	42.4	
Weeks							
≤8	1	-0.15(-0.44- 0.14)	0.12	0.309	–	–	0.109
8–12	11	-0.44(-0.64–0.25)	1.18	0.053	0.981	44.9	
>12	3	-0.08(-0.46–0.30)	0.36	0.417	<0.01	0.0	
Training intensity,%							
75–79	4	-0.35(-0.68–0.01)	0.70	0.043	0.085	54.8	0.253
80–89	6	-0.21(-0.41–0.03)	0.49	0.025	0.857	0.0	
≥90	3	-0.73(-1.21–0.26)	1.60	<0.01	<0.01	81.9	
Age, year							
<40	2	-0.21(-0.55- 0.14)	0.31	0.242	0.989	0.0	0.649
40–60	10	-0.38(-0.60–0.16)	1.04	0.015	<0.01	56.3	
≥60	3	-0.27(-0.52–0.01)	0.37	0.240	0.582	29.9	
Disease duration, year							
<5	6	-0.47(-0.75–0.19)	1.10	<0.01	<0.01	74.2	0.362
5–10	7	-0.22(-0.42–0.02)	0.48	0.028	0.948	0.0	
>10	2	-0.36(-0.77- 0.05)	0.44	0.085	0.263	20.0	
Average Duration, min/time							
≤30	11	-0.32(-0.49–0.16)	0.88	<0.01	0.024	51.4	0.320
>30	2	0.12(-0.48- 0.72)	0.35	0.698	0.303	5.6	
HbA1c							
Overall	30	-0.60(-0.84–0.36)	2.69	<0.01	<0.01	69.4	
Weeks							
≤8	5	-1.18(-1.51–0.84)	2.33	<0.01	<0.01	76.9	<0.01
8–12	19	-0.40(-0.56–0.24)	1.51	<0.01	<0.01	57.6	
>12	6	-0.68(-0.99- -0.38)	1.36	<0.01	<0.01	71.5	
Training intensity,%							
75–79	5	-0.67(-1.00–0.35)	0.87	<0.01	<0.01	73.2	0.549
80–89	15	-0.49(-0.66–0.32)	1.72	<0.01	<0.01	74.7	
≥90	5	-0.61(-0.96- -0.25)	1.18	<0.01	0.071	53.7	
Age, year							
<40	6	-0.95(-1.24–0.66)	1.98	<0.01	<0.01	68.0	<0.01
40–60	18	-0.54(-0.70–0.16)	1.83	<0.01	<0.01	65.4	
≥60	6	-0.24(-0.56–0.37)	0.66	0.138	<0.01	74.2	
Disease duration, year							
<5	8	-0.73(-0.99–0.48)	1.65	<0.01	0.107	40.7	<0.01
5–10	13	-0.39(-0.57–0.21)	1.21	<0.01	<0.01	72.2	
>10	3	-0.32(-0.70–0.05)	0.50	0.092	0.269	23.9	

**Figure 6:**
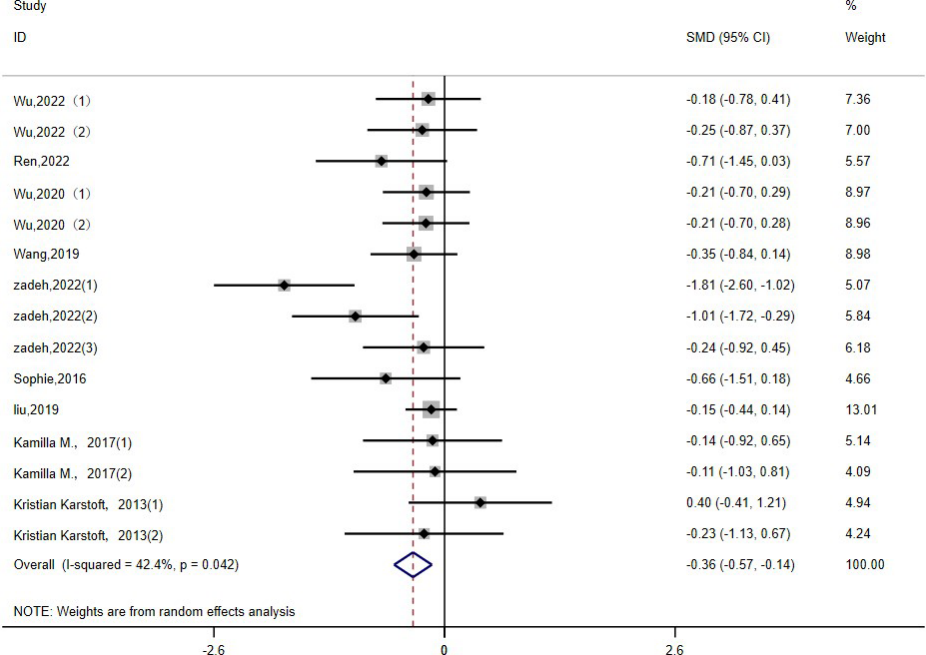
2h-PG.

2h-PG: Fifteen studies (15, 16, 19, 22, 23, 26, 30, 36, 39) evaluated the effect of HIIT on 2h-PG metrics in a type 2 diabetic population. The results show that HIIT had a large effect size on 2h-PG (MD: -0.36; 95% CI: -0.57- -0.14; Hedges’ g = 1.05; p<0.01) (**Figure 6**). Because the I^2^ was 42.4%, a random-effects model was chosen for analysis. There were no statistically significant differences in the 2h-PG results for the exercise cycle, intensity, duration and disease duration subgroups (p = 0.10, 0.25, 0.32, 0.36, respectively) (**Table 3**). However, the results were significant for exercise cycles of 8–12 weeks (p = 0.05), exercise intensities of ≥90% (p<0.01), and exercise durations of ≤30 minutes (p <0.01); all had large effect sizes and a good effect on reducing 2h-PG. The effect of 2h-PG reduction was more pronounced for a disease duration of <5 years.

**Figure 7:**
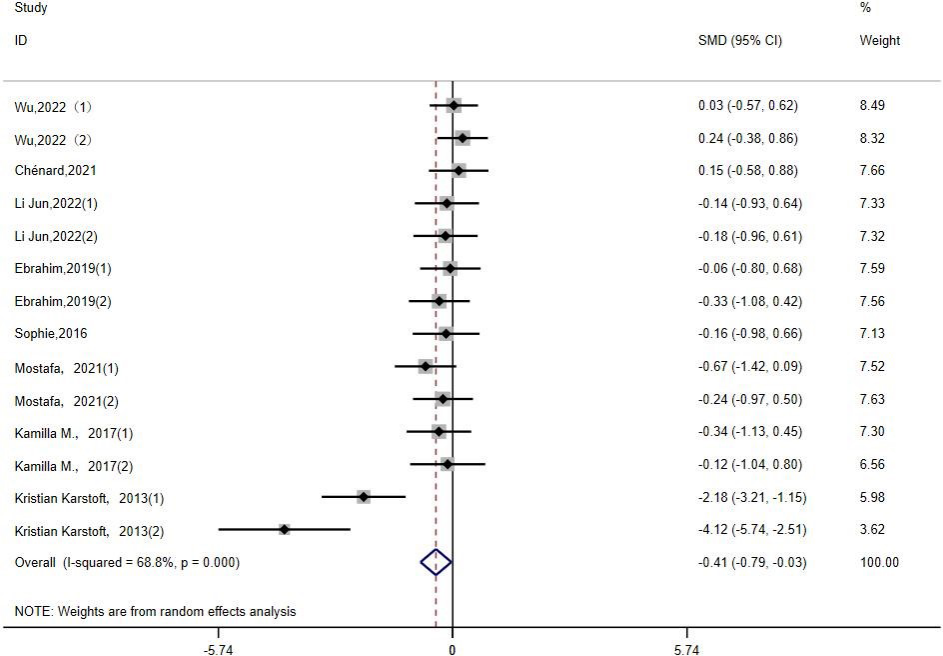
FINS.

FINS: Fourteen studies (14, 15, 18, 21–23, 30, 35) evaluated the effect of HIIT on FINS metrics in a type 2 diabetes population. The results show that HIIT had a FINS- lowering effect (MD: -0.41; 95% CI: -0.79- -0.03; Hedges’ g =1.07; p<0.01) (**Figure 7**). Because the I^2^ was 68.8%, a random-effects model was chosen for the analyses. The subgroup results for exercise intensity and duration were significantly different (p<0.01), but their confidence intervals all overlapped (**Table 3**). Therefore, there were no significant differences among any of the above subgroups, and the FINS results were not affected by exercise intensity or duration. There was no statistically significant difference in age subgroups (p = 0.447), but the results showed a significant effect of HIIT in reducing FINS in the 40–60 year olds (p < 0.01). F or people over 60 years old, the effect was not significant (p = 0.972).

**Figure 8:**
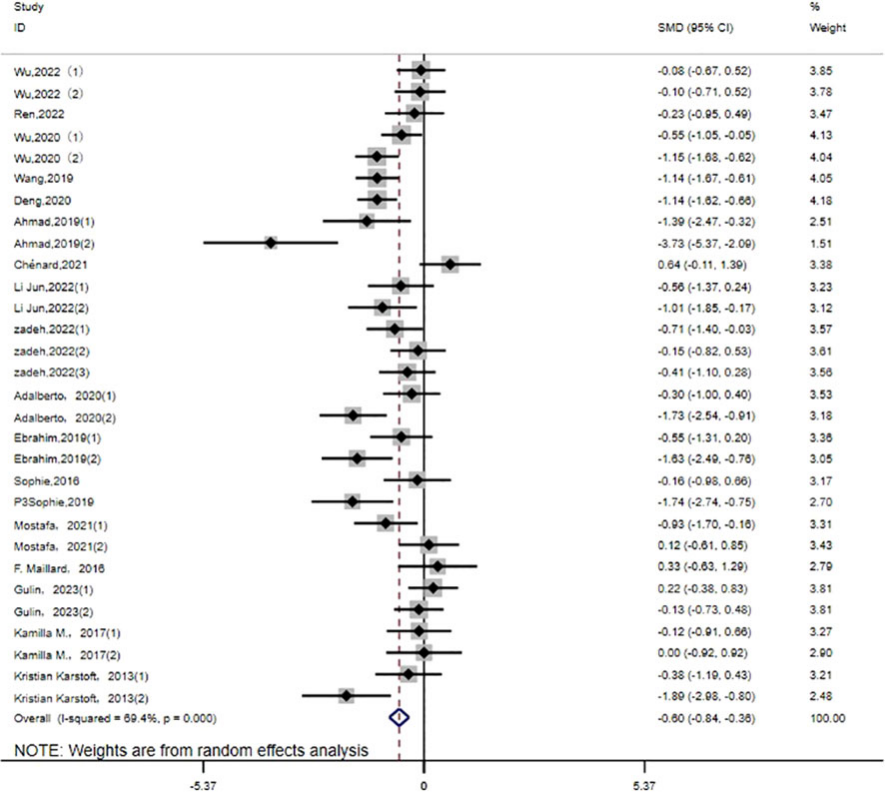
HbA1c.

HbA1c: Thirty studies (14–27, 30–32, 35) evaluated the effect of HIIT on HbA1c metrics in a type 2 diabetic population. The results show that HIIT had a large effect size on HbA1c (MD: -0.60; 95% CI: -0.84- -0.36; Hedges’ g = 2.69; p<0.01) (**Figure 8**). Because the I^2^ was 69.4%, a random-effects model was chosen for analysis. There were significant differences in the exercise cycle subgroups (**Table 3**), with larger effect size results for ≤8 weeks compared to 8–12 weeks and >12 weeks (p < 0.01). There were no significant differences in the subgroup results for exercise intensity, age or disease duration, so the HbA1c results were not affected by these. From the results, it can be concluded that the group with an age of < 40 years (p < 0.01) had a large effect size, and the results were greater for exercise intensity of 80%-89%. HbA1c reduction was more pronounced for disease duration of <5 years.

### 3.4 Secondary outcomes (lipid metabolism)

**Figure 9:**
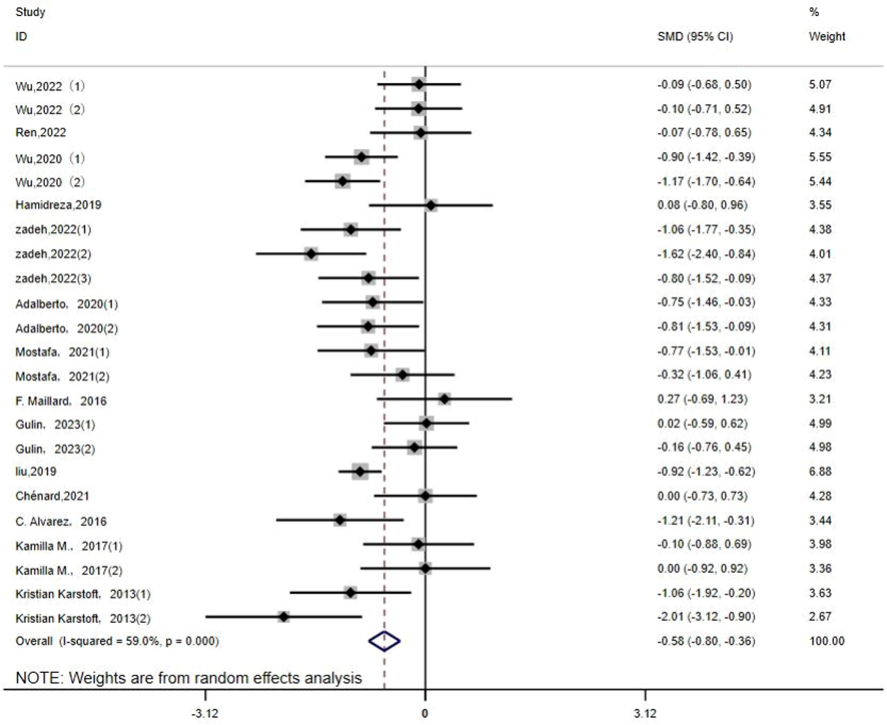
TC.

TC: Twenty-three studies (14–16, 19, 20, 22–25, 29, 32, 33, 35) assessed the effects of HIIT on markers of TC in a type 2 diabetic population. The results showed that HIIT had a large effect size on TC (MD: -0.58; 95% CI: -0.80- -0.36; Hedges’ g =2.36; p<0.01) (**Figure 9**). Because the I^2^ was 59%, a random-effects model was chosen for analysis. There was a significant difference in the subgroup results for exercise cycle and disease duration (**Table 4**), with an exercise cycle >12 weeks having a greater effect size compared to the other two subgroups. The effect size for a disease duration of less than 5 years was greater than that for the subgroup with >5 years of disease duration. However, there were no significant differences between the exercise intensity and age subgroups (p = 0.175, 0.228) and the TC values were not affected by the intensity of exercise or age, but the results show that the exercise intensity 75%-79% and 80%-89% both had large effect sizes.

**Table 4:** Subgroup analysis of lipid metabolism.

Group standard	Study quantity	Mean difference (95% CI)	Hedges’ g	P within group	Heterogeneity		
					P heterogeneity	I2 (%)	P between sub-groups
TC							
Overall	23	-0.58(-0.80–0.36)	2.36	<0.01	<0.01	59.0	
Weeks							
≤8	4	-0.81(-1.06–0.56)	1.14	<0.01	0.211	33.6	<0.01
8–12	12	-0.40(-0.60–0.21)	1.17	<0.01	0.034	56.5	
>12	7	-0.90(-1.18–0.60)	1.71	<0.01	<0.01	56.0	
Training intensity,%							
75–79	4	-1.11(-1.56–0.66)	1.51	<0.01	<0.01	75.3	0.175
80–89	11	-0.56(-0.73–0.40)	1.46	<0.01	<0.01	62.8	
≥90	4	-0.62(-1.03- -0.21)	0,72	<0.01	0.088	54.2	
Age, year							
<40	3	-0.87(-1.21–0.52)	0.94	<0.01	0.056	65.2	0.228
40–60	14	-0.52(-0.70–0.16)	0.35	<0.01	<0.01	59.8	
≥60	6	-0.65(-0.88–0.43)	0.22	<0.01	0.028	60.1	
Disease duration, year							
<5	7	-1.20(-1.48–0.92)	2.22	<0.01	0.597	0.0	<0.01
5–10	9	-0.46(-0.65–0.27)	0.99	<0.01	0.024	54.7	
>10	4	-0.37(-0.71–0.03)	0.70	0.031	0.057	60.2	
TG							
Overall	23	-0.50(-0.86–0.14)	1.50	<0.01	<0.01	83.9	
Weeks							
≤8	4	-0.33(-0.57–0.08)	0.62	<0.01	<0.01	84.8	0.715
8–12	12	-0.40(-0.60–0.20)	1.21	<0.01	<0.01	85.7	
>12	7	-0.49(-0.78–0.19)	0.52	<0.01	<0.01	84.6	
Training intensity,%							
75–79	4	-0.33(-0.86–0.19)	0.93	0.208	<0.01	95.6	<0.01
80–89	11	-0.22(-0.38–0.06)	0.66	<0.01	0.01	56.9	
≥90	4	-1.03(-1.47–0.59)	1.01	<0.01	<0.01	84.5	
Age, year							
<40	3	-0.73(-1.06–0.39)	1.09	<0.01	0.894	0.0	0.012
40–60	14	-0.48(-0.68–0.27)	1.12	<0.01	<0.01	87.6	
≥60	6	-0.15(-0.38- 0.07)	0.44	0.173	<0.01	77.6	
Disease duration, year							
<5	7	-1.20(-1.05–0.45)	1.27	<0.01	<0.01	93.2	<0.01
5–10	9	-0.20(-0.38–0.01)	0.44	0.038	0.043	54.7	
>10	4	-0.18(-0.51- 0.16)	0.40	0.304	0.048	60.2	
Average Duration, min/time							
≤30	15	-0.36(-0.52–0.20)	0.84	<0.01	<0.01	87.7	0.014
>30	6	-0.70(-0.99–0.40)	1.31	<0.01	0.090	47.5	
HDL							
Overall	24	0.62(0.29–0.95)	1.19	<0.01	<0.01	81.9	
Weeks							
≤8	4	0.42(0.17–0.67)	0.48	<0.01	<0.01	90.1	0.126
8–12	13	0.23(0.05–0.40)	0.74	0.012	0.071	39.4	
>12	7	0.57(0.26–0.89)	1.47	<0.01	<0.01	91.8	
Training intensity, %							
75–79	5	0.10(-0.21–0.42)	0.43	0.513	0.804	0.0	<0.01
80–89	11	0.23(0.07–0.40)	0.88	<0.01	<0.01	82.7	
≥90	4	0.88(0.43–1.32)	0.63	<0.01	<0.01	90.0	
Age, year							
<40	3	0.36(0.04–0.69)	0.57	0.027	0.475	0.0	0.607
40–60	15	0.28(0.09–0.46)	0.99	<0.01	<0.01	84.4	
≥60	6	0.43(0.20–0.65)	0.80	<0.01	<0.01	85.5	
Disease duration, year							
<5	7	0.52(0.25–0.79)	0.78	<0.01	<0.01	83.0	<0.01
5–10	10	0.13(-0.04–0.31)	0.67	0.136	<0.01	82.3	
>10	4	0.37(0.03- 0.70)	0.63	0.034	0.125	47.7	
LDL							
Overall	24	-0.31(-0.56–0.08)	0.91	<0.01	<0.01	67.6	
Week							
≤8	4	-0.22(-0.46- 0.03)	0.11	0.080	0.035	65.1	<0.01
8–12	13	-0.06(-0.23- 0.12)	0.19	0.515	0.861	0.0	
>12	7	-0.66(-0.96–0.36)	0.98	<0.01	<0.01	86.4	
Training intensity, %							
75–79	5	-0.26(-0.60–0.08)	0.14	0.132	<0.01	91.0	0.422
80–89	11	-0.15(-0.31–0.01)	0.36	0.073	0.206	24.9	
≥90	4	-0.22(-0.62–0.18)	0.31	0.275	0.200	35.4	
Age, year							
<40	3	-0.19(-0.51–0.13)	0.22	0.250	0.964	0.0	0.799
40–60	15	-0.25(-0.44–0.07)	0.51	<0.01	<0.01	73.7	
≥60	6	-0.16(-0.38–0.06)	0.39	0.157	<0.01	71.1	
Disease duration, year							
<5	7	-0.55(-0.83–0.28)	0.78	<0.01	<0.01	82.5	<0.01
5–10	10	-0.07(-0.25–0.31)	0.12	0.416	0.240	22.0	
>10	4	-0.05(-0.39- 0.10)	0.15	0.777	0.044	62.9	
Average Duration, min/time							
≤30	15	-0.14(-0.30–0.01)	0.41	0.065	0.430	1.8	0.121
>30	6	-0.50(-0.81–0.20)	0.79	<0.01	0.090	88.4	

**Figure 10:**
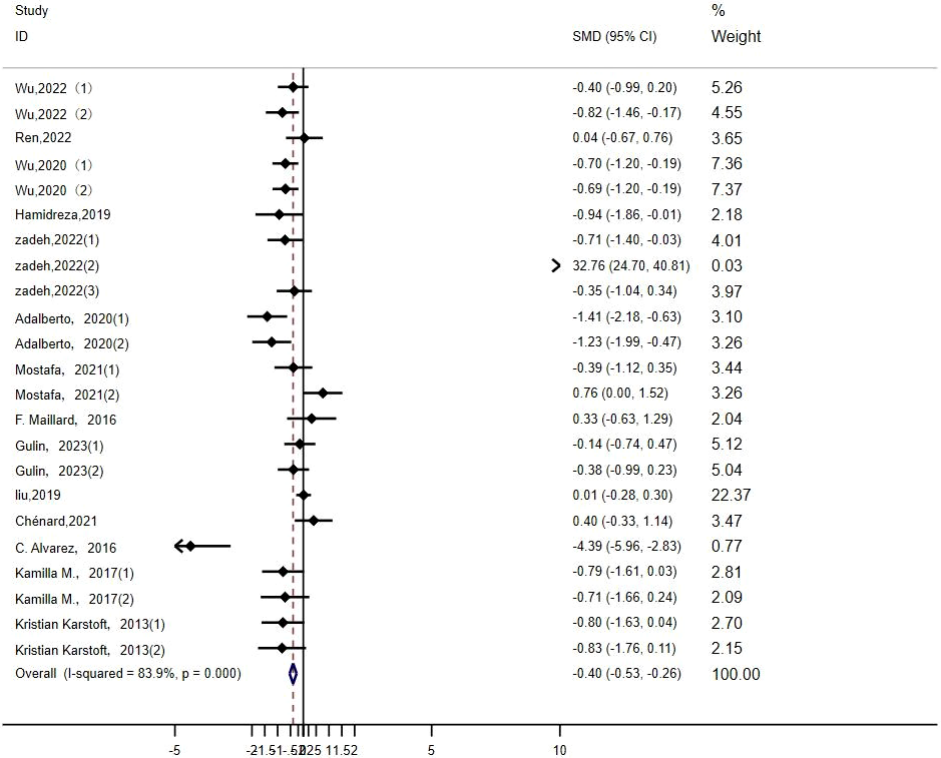
TG.

TG: Twenty-three studies (14–16, 19, 20, 22–25, 29, 32, 33, 35) evaluated the effect of HIIT on markers of TG in type 2 diabetic population. We found that there was a large effect size of HIIT intervention on TG (MD: -0.50; 95% CI: -0.86- -0.14; Hedges’ g =1.50; p<0.01) (**Figure 10**). Because the I^2^ was 83.9%, subgroup analyses were performed for the exercise period, intensity, duration, age, and disease duration. There were significant differences in the exercise duration subgroups (**Table 4**), with durations >30 min reducing TG values more than durations ≤30 min. There were no significant differences between the subgroups for the remaining groups, and the TG values were not affected by the exercise cycle, intensity, age, or disease duration. An exercise cycle of 8 to 12 weeks was found to have a large effect size. An age of 40–60 years (p<0.01) had a better effect size for exercise, and an age of >60 years had no significant effect size for exercise (p=0.173). The effect sizes were larger for illness durations of less than 5 years.

**Figure 11:**
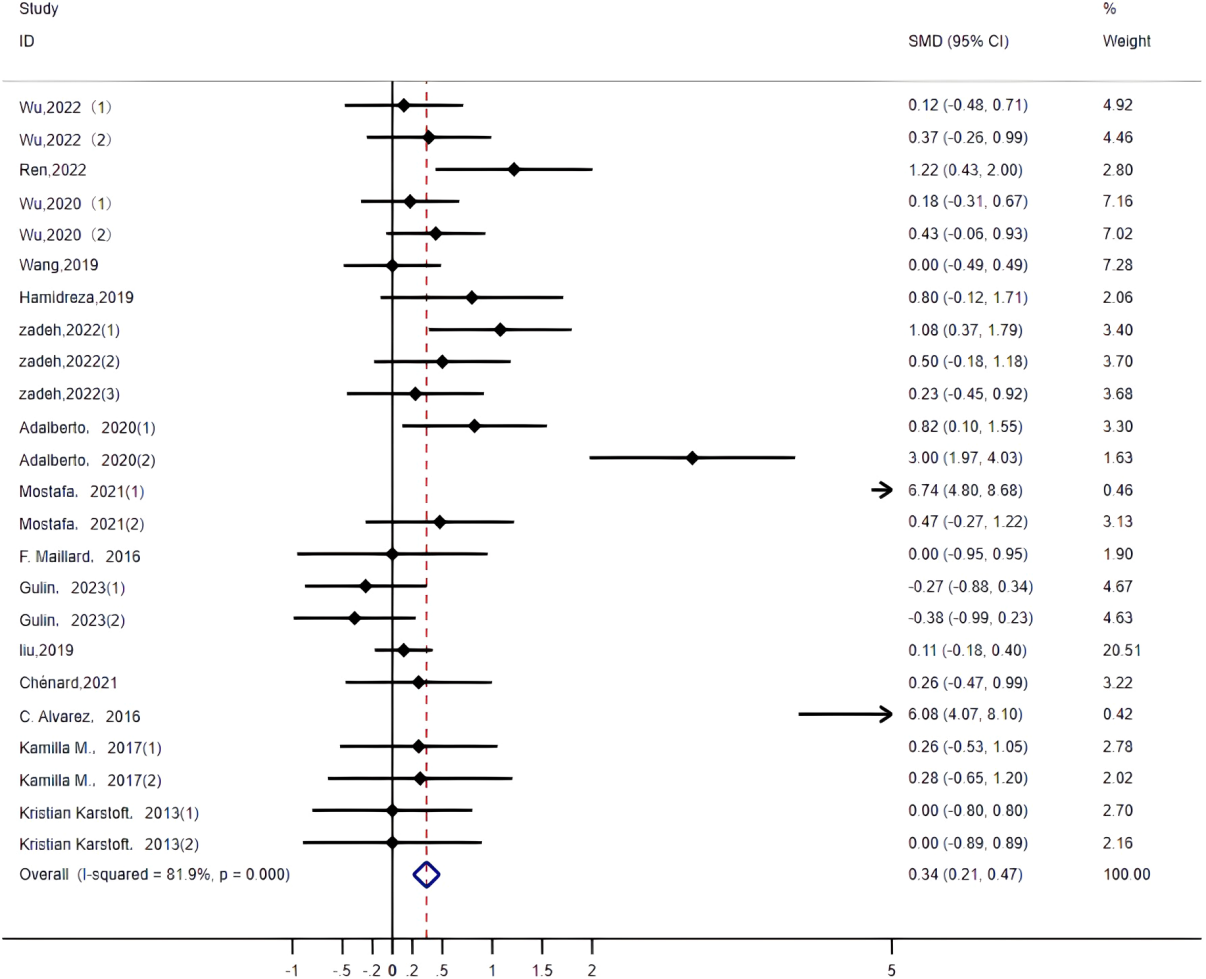
HDL.

HDL: Twenty-four studies (14–16, 19, 20, 22–26, 29, 32, 33, 35, 36) assessed the impact of HIIT on HDL indicators in a type 2 diabetes population. The r esults showed that HIIT promoted HDL with a large effect size (MD: 0.62; 95% CI: 0.29–0.95; Hedges’ g =1.19; p<0.01) (**Figure 11**). Because the I^2^ was 81.9%, a random-effects model was chosen for analysis. Although the differences between the subgroups for exercise intensity and disease duration were significant (p<0.01) (**Table 4**), there was an overlap in the confidence intervals, so none of the above subgroups were significantly different, suggesting that the HDL results were not affected by the exercise cycle, intensity, age or disease duration. The results show that exercise cycles of >12 weeks were more effective in promoting HDL in people than exercise cycles of ≤ 8 weeks. Disease durations of less than 5 years had a large effect size. An exercise intensity of 80%-89% (p<0.01) had a large effect size. The group aged 40–60 (p<0.01) and the group over 60 (p<0.01) were both highly effective groups, with greater contributions to HDL.

**Figure 12:**
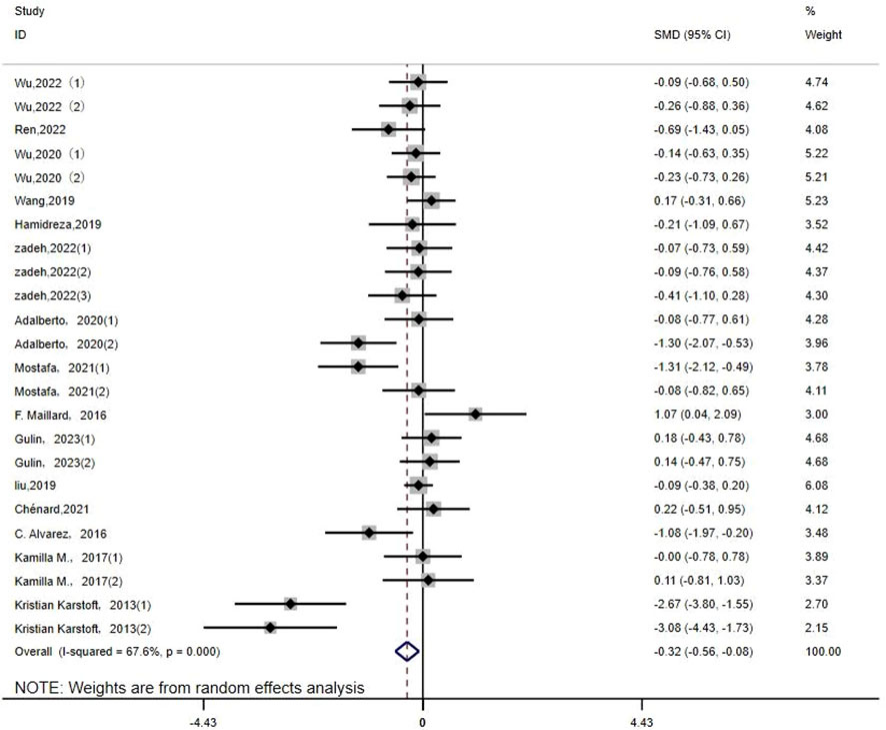
LDL.

LDL: Twenty-four studies (14–16, 19, 20, 22–26, 29, 32, 33, 35, 36) assessed the impact of HIIT on LDL metrics in a type 2 diabetes population. The results showed that HIIT had a large effect size on LDL (MD: -0.31; 95% CI: -0.56–0.08; Hedges’ g = 0.91; p<0.01) (**Figure 12**). Because the I^2^ was 67.6%, a random-effects model was chosen for analysis. Although there was a significant difference between the exercise cycle and disease duration subgroups (p<0.01) (**Table 4**), the confidence intervals overlapped, so there was no significant difference between the subgroups, suggesting that the LDL results were not influenced by the exercise cycle, intensity, duration, age or disease duration. The results show that an exercise period of >12 weeks (p<0.01) had a large effect size. Exercise durations of >30 min (p<0.01) had a better exercise effect. An age of 40–60 years of age (p<0.01) significantly reduced LDL values with moderate effect size. A disease duration of <5 years (p<0.01) had a large effect size.

### 3.5 Sensitivity analysis

Sensitivity analysis of the findings for each indicator showed that the fasting glucose indicator produced a large bias due to the removal of the article by Deng, 2020 (27); the HDL indicator produced a large bias due to the removal of the articles by Adalberto, 2020(2) (20), Mostafa, 2021(1) (14), and Alvarez, 2016 (33); the 2h-PG indicator produced a large bias due to the removal of the articles by Zadeh, 2022 (19); and the FINS indicator produced a large bias due to the removal of the articles by Kamilla, 2017(1) (23) and Kamilla, 2017(2) (23). Therefore, the combined results of the above indicators may be unstable, and the combined results of the other indicators may be more stable **(Annex 1)**.

### 3.6 Publication bias

We examined the publication bias for each indicator based on the funnel plot analysis. It was found that the study sites were found to be basically distributed on either side of the x=0 vertical line, but there was still a small number of study sites scattered regarding the FPG index, LDL index, and TC index. This suggests that there may be some publication bias (**Annex 2**).

## 4 Discussion

There is now a growing body of literature suggesting that the HIIT exercise model is more compatible with people undertaking self-directed interventions, that it is more feasible relative to MICT, and that it helps to improve exercise adherence (13, 40). Additionally, there is new evidence for its effects on human health (40–43). The latest physical activity guidelines emphasize, for the first time, the value of intermittent short-duration physical activity in building up the recommended amount of physical activity. Achieving weekly vigorous physical activity (VPA) goals with 10 minutes of VPA 3–4 times a day, 2–3 days a week, is beneficial for glycolipid metabolism and can increase life expectancy (41). Mengyun Luo et al. noted that longer durations of exercise are associated with a lower risk of T2DM, and that sustained exercise of 68.4 minutes or more reduced the incidence of T2DM by approximately 74% (42). Dong Hoon Lee et al. found that when exercise time was increased to two to four times the amount recommended by the World Health Organization, i.e., 150–299 minutes of VPA per week, the participants experienced a 21–23% reduction in all-cause mortality, a 27–33% reduction in cardiovascular disease mortality, and a 19% reduction in non-cardiovascular disease mortality (43).

Previous studies (6, 9, 10) have not reached consistent conclusions on whether HIIT improves glycolipid metabolism in patients with type 2 diabetes and there is a lack of data from studies involving HIIT intervention protocols (e.g., training intensity, training frequency, and total duration) and patient characteristics (age, duration of diabetes). To evaluate this type of exercise and obtain higher-level evidence, we performed this meta-analysis.

The results from this study indicate that HIIT has a positive effect on glucose-lipid metabolism in patients with type 2 diabetes mellitus. In terms of glucose metabolism, for the FBG, 2h-PG, and HbA1c indexes, HIIT was more advantageous than MICT for lowering their levels. In terms of lipid metabolism, HIIT was more favorable than MICT for in lowering TC, and there were no significant difference in the rest of the indicators. Furthermore, the results of the study show that an exercise program with a medium to long duration (> 8 weeks) and medium to high intensity (80%-89%) had a greater effect on most glycolipid indices. An exercise duration of > 30 min was more effective in lowering lipid indices, while sustained exercise for ≤ 30 min had a significant effect on lowering blood glucose levels. The majority of the indicators showed no significant effect of exercise in people > 60 years of age, but for the HDL indicator, people aged > 60 years of age had increased HDL values instead.

The effect sizes of the findings indicate that HIIT can reduce four types of glycemic indicators: FPG, 2h-PG, FINS, and HbA1c. Due to the high heterogeneity, we analyzed subgroups of relevant information for each indicator. In all age subgroups, the glycemic- lowering effect of HIIT was not significant for those aged over 60 years, and those aged 40–60 years were able to obtain more benefits from exercise training. All disease duration subgroups demonstrated that those with less than 5 years of disease duration could obtain better exercise results from HIIT than those with more than 5 years of disease. The r esults showed that a moderate-to-high intensity, moderate-to-long duration exercise program had a more significant effect on lowering blood glucose. For lowering HbA1c, the effect size results were greater for exercise cycles of <8 weeks and exercise intensities of 80%-89% or even higher. Previous studies (15, 26, 37) have shown HIIT to be effective in improving fasting blood glucose and HbA1c levels, and to facilitating glycemic control compared to other exercises. Winding et al. showed that HbA1c, fasting blood glucose, postprandial blood glucose, glycemic variability, and HOMA-IR were all reduced after HIIT (23). The results of the present study are consistent with the results of previous studies.

HbA1c levels are closely related to microvascular complications in diabetes, with studies showing that a 1% reduction in HbA1c levels is associated with a 14% reduction in myocardial infarction rates and a 21% reduction in the risk of diabetes-related death (12, 14). Therefore, HbA1c is an important indicator for evaluating diabetes therapies. The type, intensity and volume of exercise affect the degree of reduction in HbA1c levels (12). The results of this study suggest that HIIT with exercise cycles of <8 weeks and moderate intensity is more effective in reducing HbA1c, and in previous studies (12, 38) it was concluded that short intervals, medium to long cycles (11–16 weeks), and moderate-intensity HIIT exercise regimens were more beneficial for patients with T2DM. HbA1c is a blood marker that quantifies the three-month average glucose concentration (12), and it may take more than 12 weeks for an exercise program to demonstrate an effect on HbA1c, but many studies have durations of 12 weeks or less. Therefore, a greater amount of the literature needs to be included and further studies should be conducted to demonstrate the effects of different HIIT programs on HbA1c.

The results of this study show that HIIT can effectively reduce the three types of lipid metabolic indicators—TC, TG, and LDL— while increasing HDL indexes. Feng Chen et al. found that HIIT can reduce TC, TG, and LDL-C levels while increasing HDL-C levels in patients (44). Yajing et al. noted that patients’ glycemic and lipid indices were significantly improved after implementing a HIIT exercise program (26). The results of this study are consistent with the above studies. Due to the high heterogeneity, we performed subgroup analyses with relevant information (exercise cycle, intensity, duration, disease duration, and age) for each indicator. The results show that exercise regimens with a long cycle (>12 weeks), moderate to high intensity (80%-89% or ≥90%), and >30 min duration had more pronounced effects on lipid indicators, and that people aged 40–60 years and with a disease duration of less than 5 years were able to derive greater benefits from HIIT. However, for the TC values, the effect of exercise intensity of 75%-79% was shown to be more significant, and due to the small number of included studies for the relevant criteria in this paper, further research on the effect of exercise intensity on TC values is required.

The results of this study show that HIIT improves glycemic control and lipid metabolism and has more potential to facilitate the implementation of completion in the lives of patients. Comparing the experimental and control groups, the daily energy expenditure control was balanced between the two groups. Compared to MICT, HIIT exercise is more intense, but the duration of exercise is shorter, and its feasibility is higher because targeted training is easier to accomplish in a short period of time. This advantage may make HIIT an effective strategy for improving clinical application in patients. At the same time, many studies have confirmed that resistance training can also enhance the effect of insulin (39, 45), and several studies have shown that HIIT combined with resistance training may provide additional benefits for patients with T2DM; whether HIIT paired with resistance exercise has a greater improvement in type 2 diabetes mellitus or not, more studies are needed to confirm this.

Strengths and limitations: This review evaluated the interventional effects of HIIT on glycolipid metabolism in a type 2 diabetic population. However, there is still a lack of convincing studies on evaluating HbA1c as well as TC; therefore, there is a need to include more of the literature for additional studies to demonstrate the effects of different HIIT regimens on HbA1c and TC. The strengths of this meta-analysis are as follows. The first is the more systematic and complete data extraction. We searched for target studies from nine countries and in two languages (English/Chinese), which further minimized regional bias and language bias. The subjects’ personal information and the intervention programs were extracted more comprehensively. Secondly, we analyzed two methods (Hedges’ g and mean difference) for the effect of exercise training. For example, Hedges’ g reflects the actual clinical effect, while the mean difference reflects the statistical effect, thus providing a clear understanding of the impact of different factors on the intervention. The optimized plan can provide personalized exercise prescriptions for T2DM patients; for example, patients aged 40–60 years and with a disease duration of less than 5 years can benefit more from HIIT. Overall, exercise programs of moderate to high intensity (80%-90% or >90%) and exercise cycles (8–12 weeks or >12 weeks) have more significant effects. The main limitation of this study is that we did not differentiate between the pre-illness and post-illness stages of the included T2DM patients, and the severity and progression of the disease may have affected the study outcomes.

## 5 Conclusions

HIIT has been shown to improve glucose and lipid metabolism in patients with type 2 diabetes, especially in HbA1c, TC, TG, and HDL. For patients between the ages of 40 and 60 with less than 5 years of disease, exercise programs of a moderate to longer duration or moderate to high intensity will produce more favorable results. Whether HIIT paired with resistance training provides greater improvement in the treatment of type 2 diabetes is subject to verification via future supplementary studies.

## Author contributions

JF: Writing – review & editing, Writing – original draft, Software, Methodology, Data curation. QZ: Writing – review & editing, Supervision, Methodology. BC: Writing – review & editing, Supervision, Methodology. JC: Writing – review & editing, Software. WW: Writing – review & editing, Software, Investigation, Data curation. YH: Writing – review & editing, Software, Investigation. JY: Writing – review & editing, Software, Methodology. HH: Writing – review & editing, Validation, Software, Project administration, Methodology, Funding acquisition.
