# Supplementary material for: Effects of high-intensity intermittent exercise on glucose and lipid metabolism in type 2 diabetes patients: a systematic review and meta-analysis
Source: Front Endocrinol (Lausanne). 2024 Jun 24;15:1360998. doi: 10.3389/fendo.2024.1360998 (PMC11229039; doi:10.3389/fendo.2024.1360998)
Supplement: Supplementary file 1 [file Image_1.pdf]

Appendix 1: Sensitivity analysis for meta-analysis

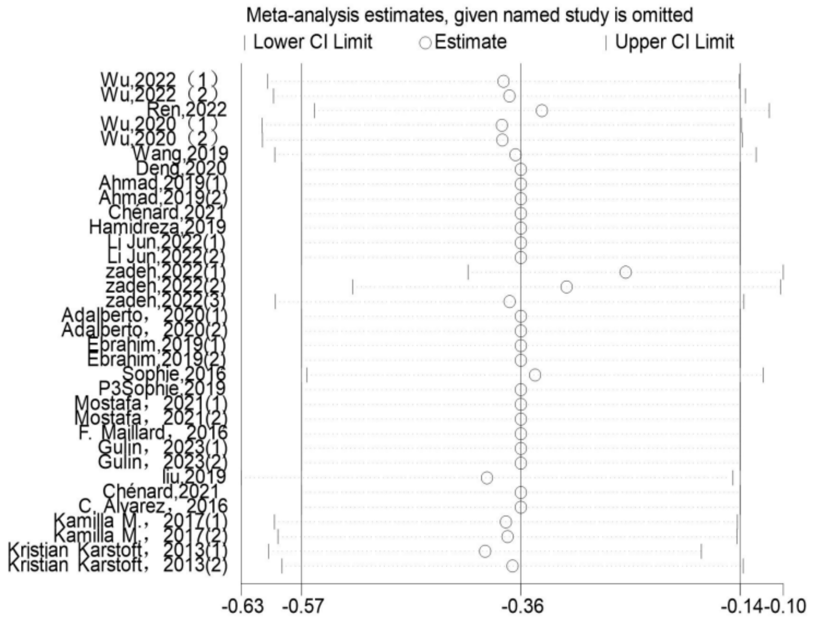

Appendix figure A. 2H-PG

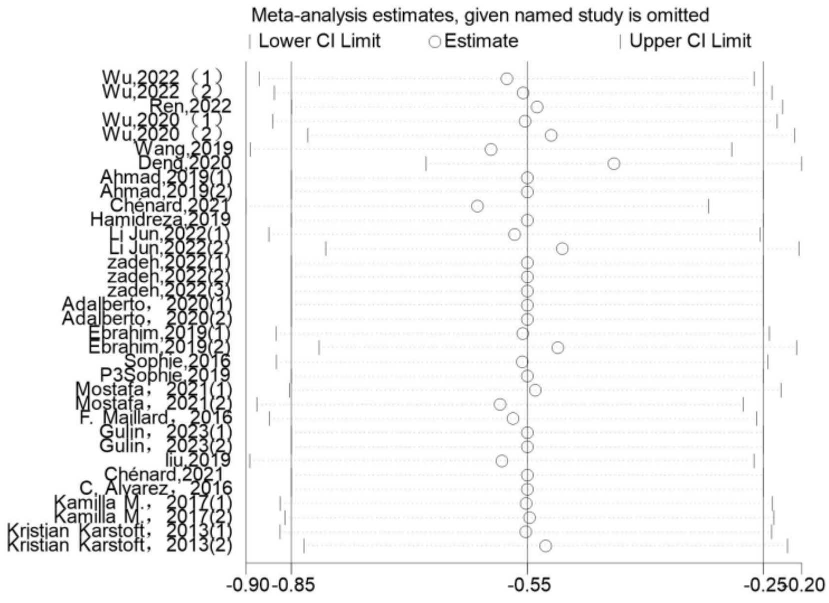

Appendix figure B. FBG

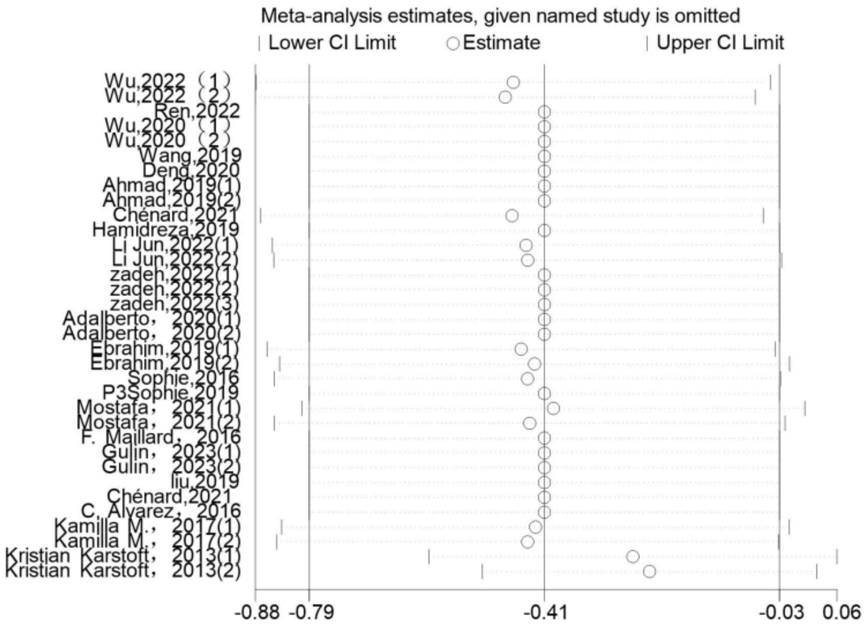

Appendix figure C. FINS

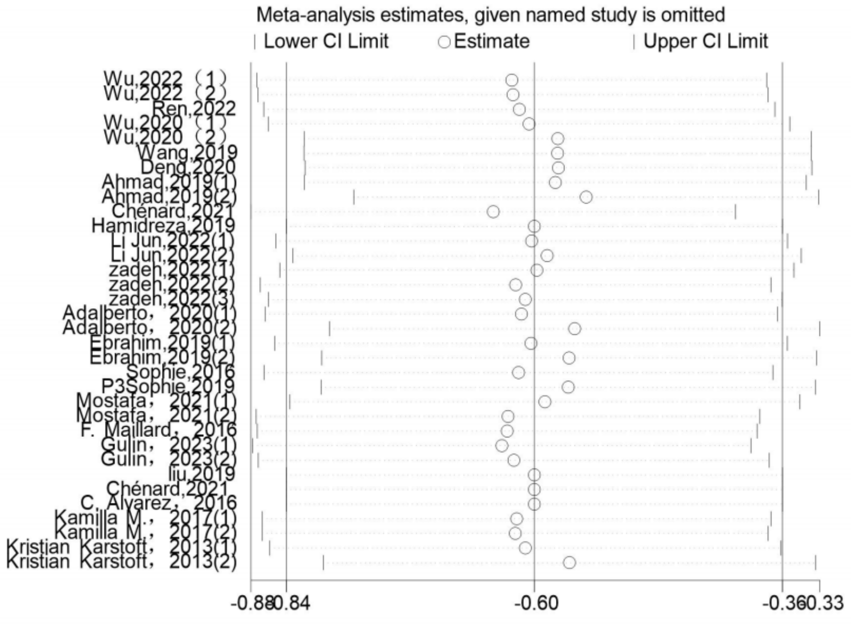

Appendix figure D. HbA1c

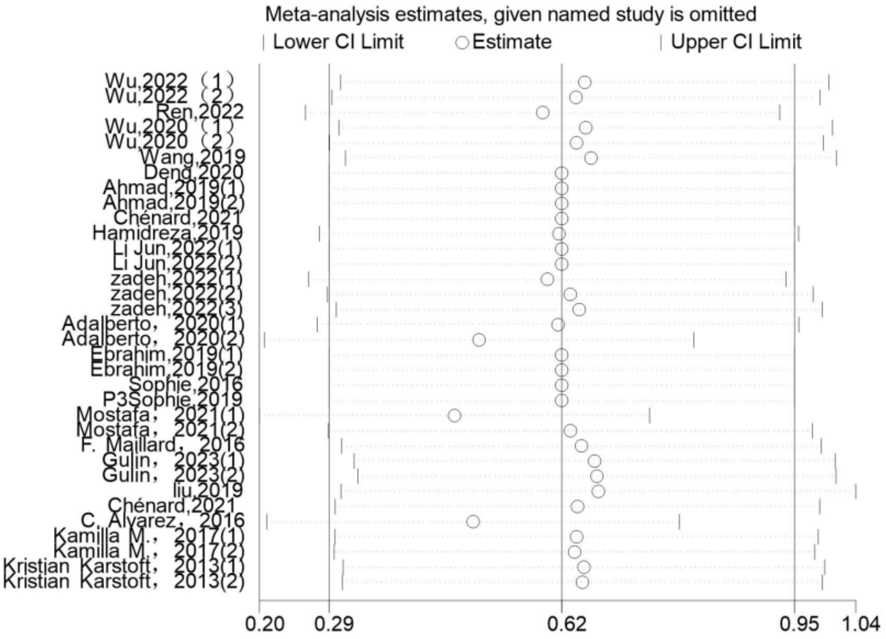

Appendix figure E. HDL

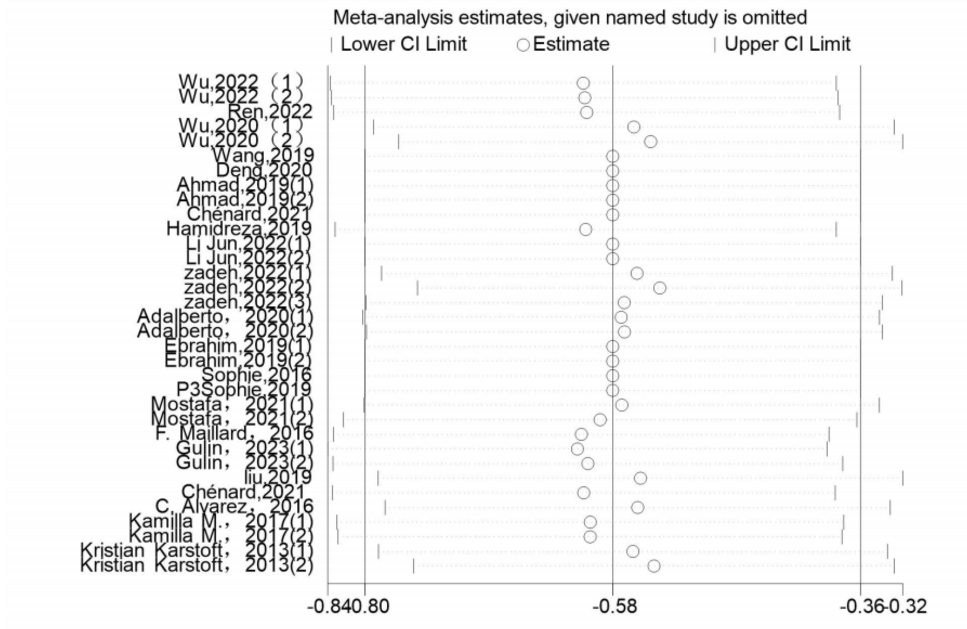

Appendix figure F. TC

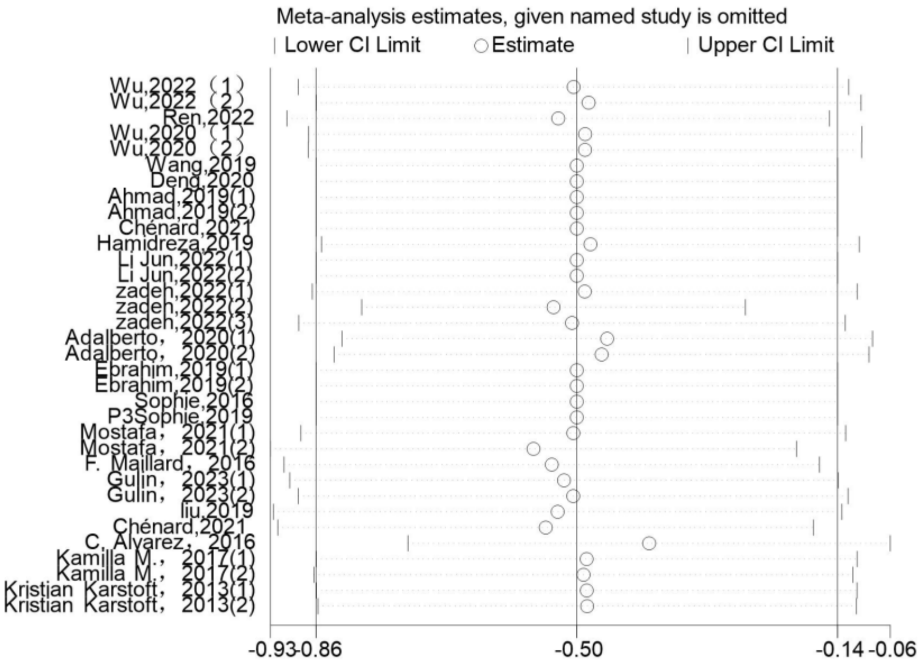

Appendix figure G. TG

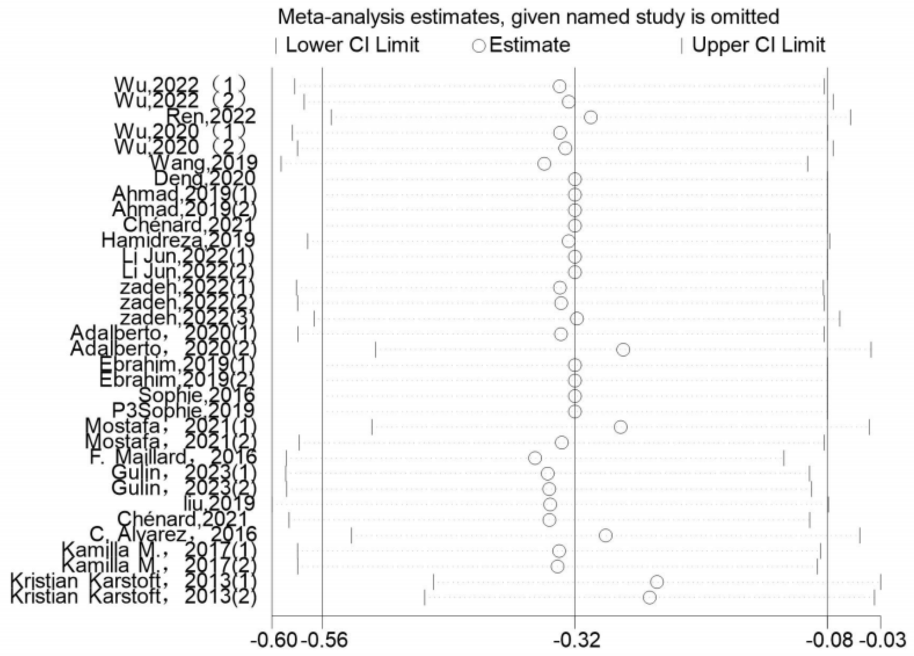

Appendix figure H. LDL

Appendix 2: Funnel plot for meta-analysis

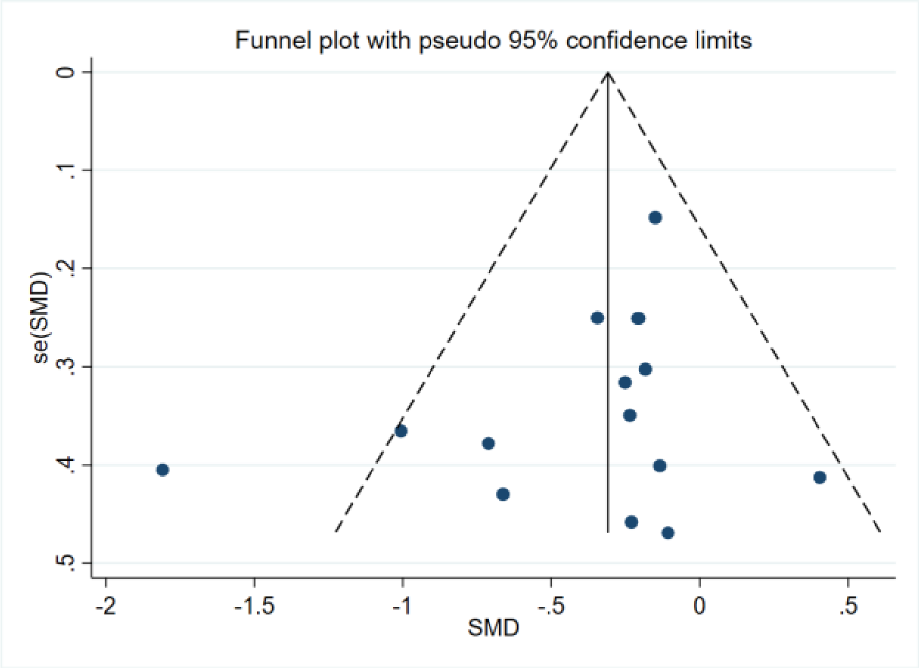

Appendix figure I. 2H-PG

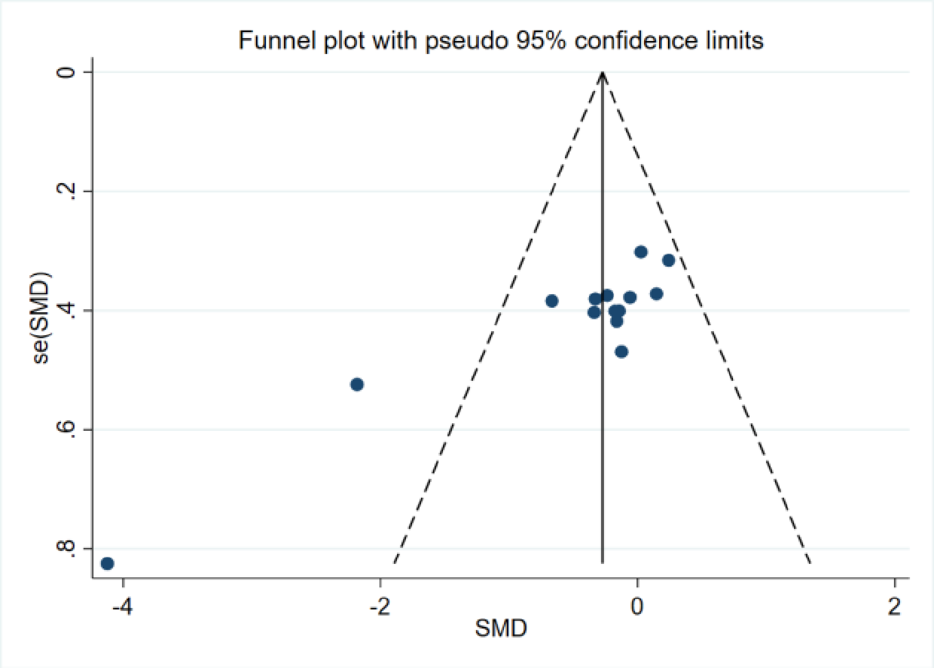

Appendix figure J. FINS

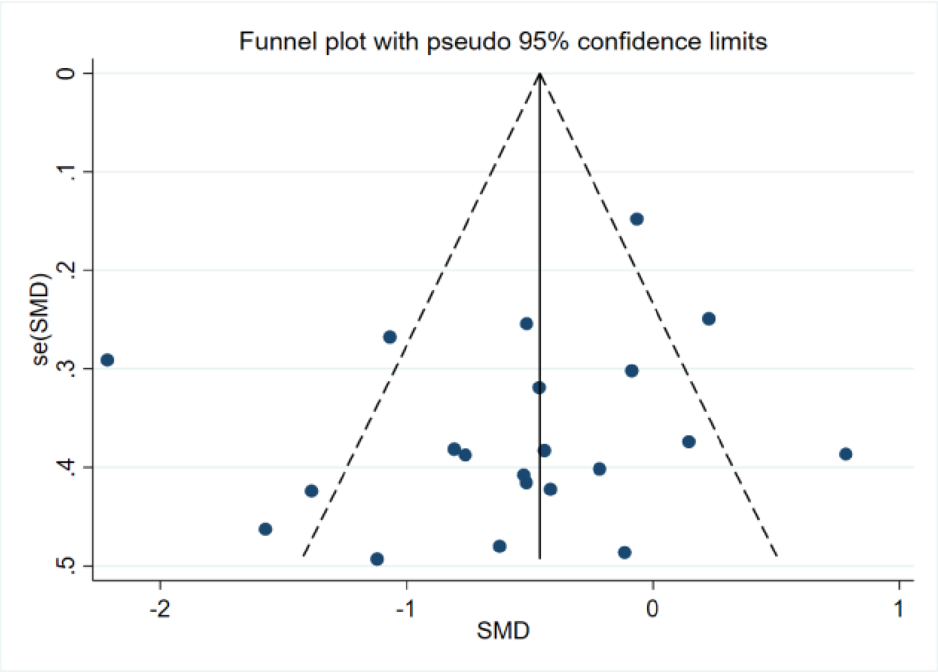

Appendix figure K. FBG

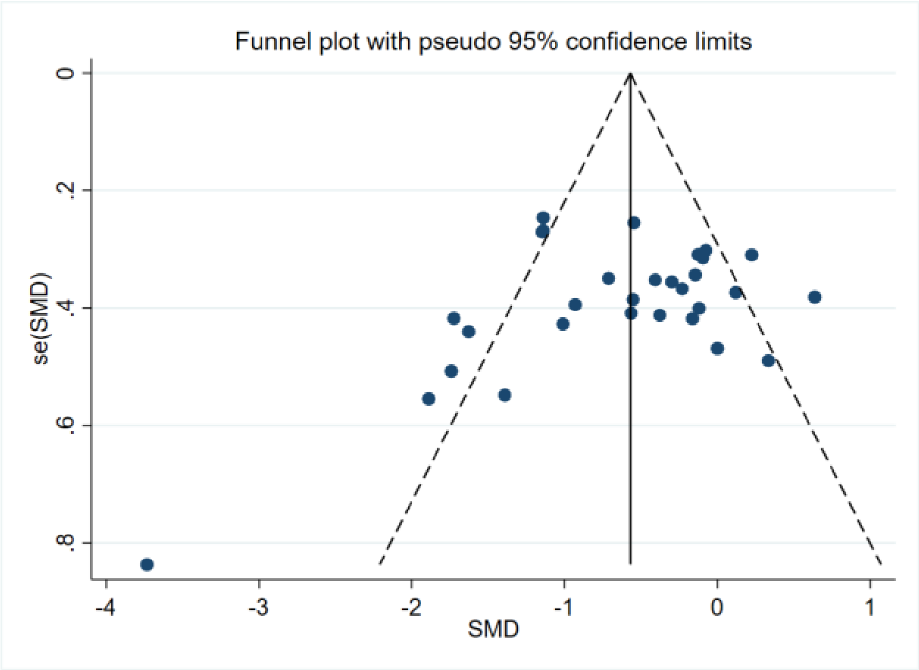

Appendix figure L. HbA1c

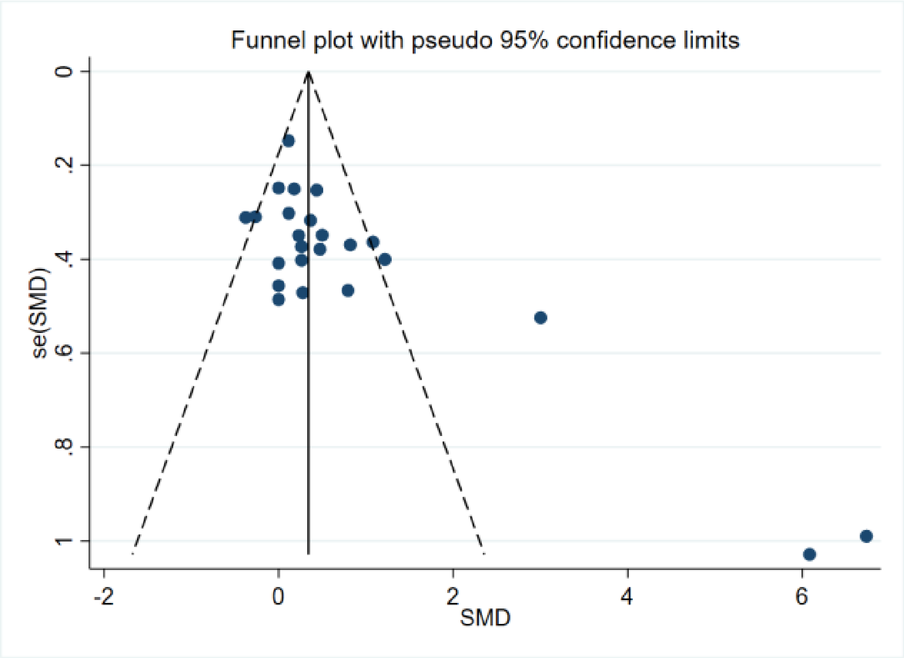

Appendix figure M. HDL

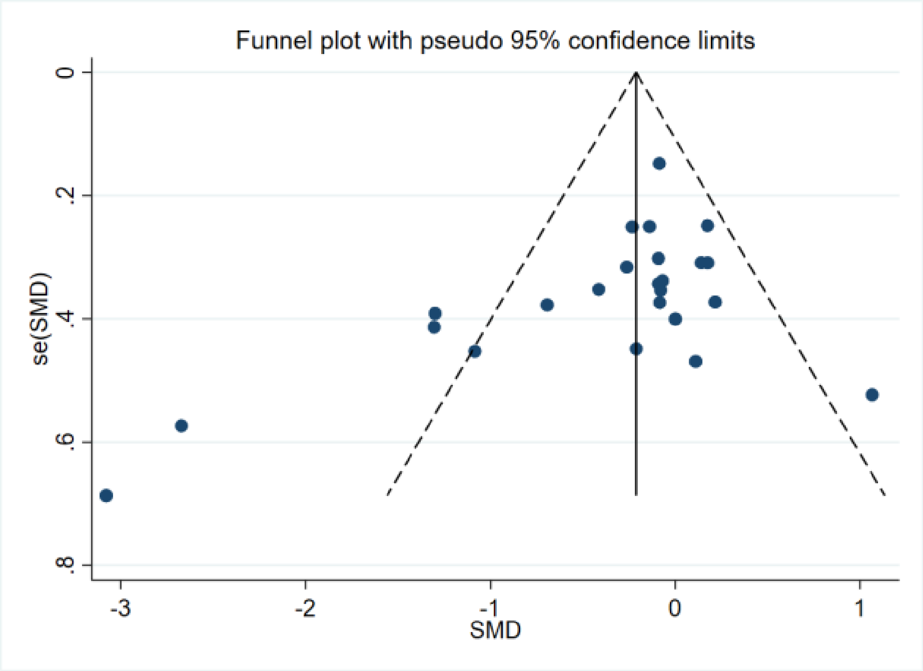

Appendix figure N. LDL

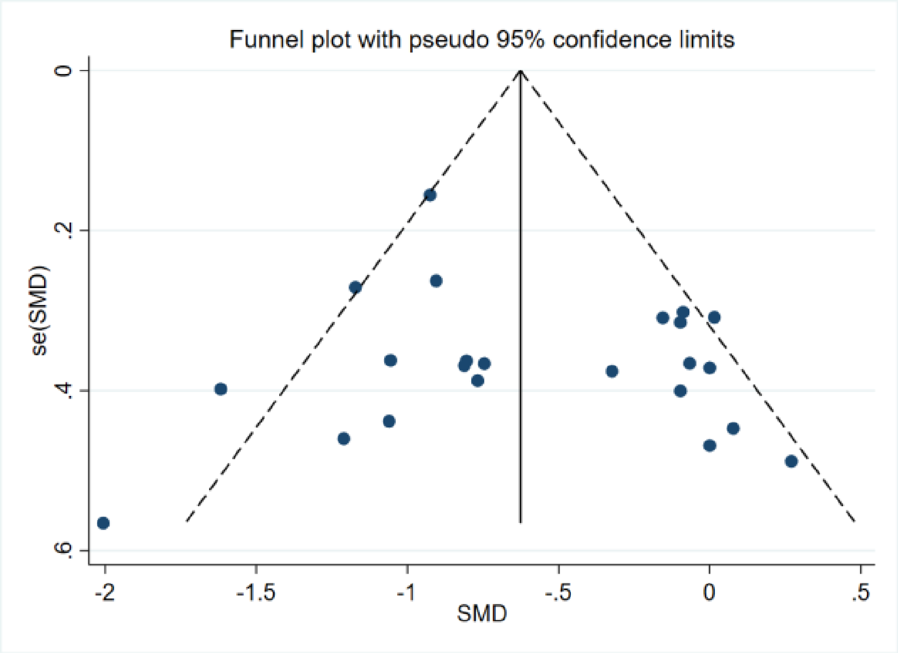

Appendix figure O. TC

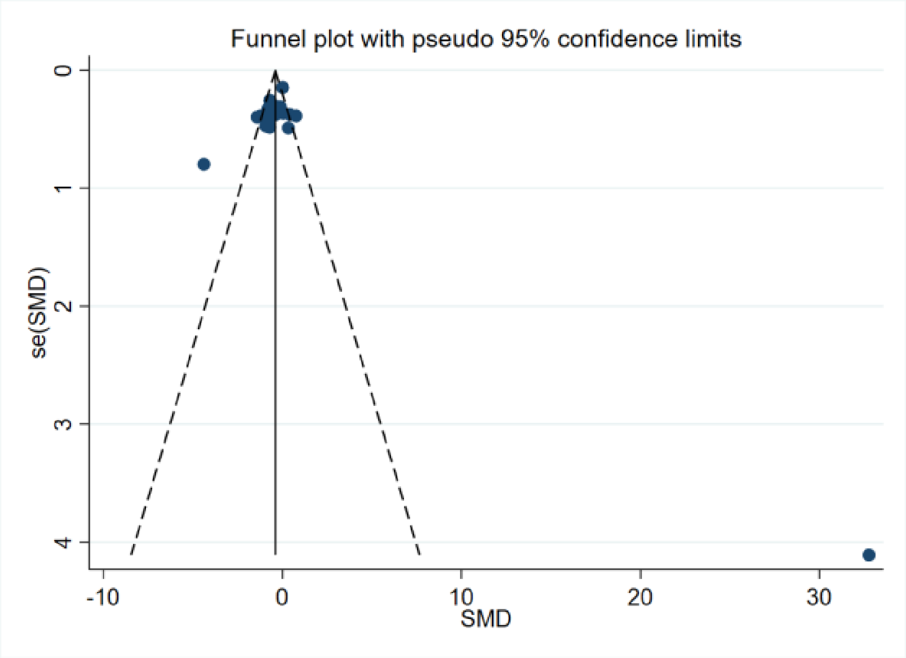

Appendix figure P. TG
